# Mechanism exploration and biomarker identification of glycemic deterioration in patients with diseases of the exocrine pancreas

**DOI:** 10.1038/s41598-024-52956-x

**Published:** 2024-02-22

**Authors:** Zhen Wang, Guolin Zhang, Jixian Fu, Guangxing Li, Zhihao Zhao, HyokChol Choe, Kaiyue Ding, Junnan Ma, Jing Wei, Dong Shang, Lin Zhang

**Affiliations:** 1 Institute (College) of Integrative Medicine, Dalian Medical University, Dalian, 116044 China; 2 Department of General Surgery, The First Affiliated Hospital of Dalian Medical University, Dalian, 116000 China; 3 Department of Cardiology II, The Second Affiliated Hospital of Dalian Medical University, Dalian, 116027 China; 4 Department of Interventional Radiology, The First Hospital of China Medical University, Shenyang, 110001 China; 5 Department of Clinical Medicine, Sinuiju Medical University, Sinuiju, Republic of Korea; 6 Department of Immunology, College of Basic Medical Science, Dalian Medical University, Dalian, 116044 China

**Keywords:** Computational biology and bioinformatics, Biomarkers, Endocrinology, Pathogenesis

## Abstract

The damage to the endocrine pancreas among patients with diseases of the exocrine pancreas (DP) leads to reduced glycemic deterioration, ultimately resulting in diabetes of the exocrine pancreas (DEP). The present research aims to investigate the mechanism responsible for glycemic deterioration in DP patients, and to identify useful biomarkers, with the ultimate goal of enhancing clinical practice awareness. Gene expression profiles of patients with DP in this study were acquired from the Gene Expression Omnibus database. The original study defines DP patients to belong in one of three categories: non-diabetic (ND), impaired glucose tolerance (IGT) and DEP, which correspond to normoglycemia, early and late glycemic deterioration, respectively. After ensuring quality control, the discovery cohort included 8 ND, 20 IGT, and 12 DEP, while the validation cohort included 27 ND, 15 IGT, and 20 DEP. Gene set enrichment analysis (GSEA) employed differentially expressed genes (DEGs), while immunocyte infiltration was determined using single sample gene set enrichment analysis (ssGSEA). Additionally, correlation analysis was conducted to establish the link between clinical characteristics and immunocyte infiltration. The least absolute shrinkage and selection operator regression and random forest combined to identify biomarkers indicating glycemic deterioration in DP patients. These biomarkers were further validated through independent cohorts and animal experiments. With glycemic deterioration, biological processes in the pancreatic islets such as nutrient metabolism and complex immune responses are disrupted in DP patients. The expression of ACOT4, B2M, and ACKR2 was upregulated, whereas the expression of CACNA1F was downregulated. Immunocyte infiltration in the islet microenvironment showed a significant positive correlation with the age, body mass index (BMI), HbA1c and glycemia at the 2-h of patients. It was a crucial factor in glycemic deterioration. Additionally, B2M demonstrated a significant positive correlation with immunocyte infiltration and clinical features. Quantitative real-time PCR (qRT-PCR) and western blotting confirmed the upregulation in B2M. Immunofluorescent staining suggested the alteration of B2M was mainly in the alpha cells and beta cells. Overall, the study showed that gradually increased immunocyte infiltration was a significant contributor to glycemic deterioration in patients with DP, and it also highlighted B2M as a biomarker.

## Introduction

The pancreas contains two compartments, the exocrine and endocrine, which are derived from the same progenitor cells. These compartments comprise 90% and 2–5% of the organ, respectively. The endocrine compartment is positioned between the exocrine compartment and both compartments work together to preserve metabolic homeostasis by releasing zymogens or hormones. Diseases of the exocrine pancreas (DP) encompass acute pancreatitis (AP), chronic pancreatitis (CP), pancreatic tumors and so on. In individuals, inflammatory responses and oxidative stress are catalysts for disturbing the pancreas’ overall structure and its metabolic endocrine balance, usually leading to a decrease in insulin release. This can result in glycemic deterioration and eventually diabetes of the exocrine pancreas (DEP)^1–3^. It has been reported^4,5^ that 45–65% of pancreatic cancer patients develop diabetes, with up to 80% experiencing new-onset hyperglycemia. A cross-sectional study^6^ revealed that 67% of CP patients had impaired glucose tolerance (IGT) or diabetes. Those with glycemic deterioration are at a higher risk of gastrointestinal and psychiatric disorders, hospitalization, and mortality compared to the average DP patient^7–11^.

Currently, research into glycemic deterioration in DP patients has mostly used retrospective clinical data analysis, with limited exploration of specific mechanisms^12,13^. This is primarily due to difficulties in identifying suitable study candidates^14^. The principal method for investigating individual glycemic deterioration mechanisms is the enzymatic isolation of pancreatic islets. However, pancreatic islets treated with digestive enzymes tend to undergo autolysis. Furthermore, patients suffering from DP usually undergo intricate treatments such as parenteral nutrition, insulin infusion, and corticosteroid therapy, which disturb the endocrine axis and modify the metabolic homeostasis of pancreatic islets^14,15^. Therefore, Barovic et al.^14,16^ established complementary platforms to harvest pancreatic tissue from DP patients who underwent pancreatectomy. The authors have demonstrated that utilizing a platform-based method for tissue collection effectively mitigates the impact of enzyme catabolism and drug therapy on the status of islets, thereby enhancing the accuracy and dependability of the data^17,18^.

Recently, Li et al.^13^ conducted a study in which they analyzed data from aforementioned platforms. The aim was to identify inflammatory biomarkers of DEP by comparing transcriptional differences between DP patients without glycemic deterioration and those with DEP. Recently, it is important to note that glycemic deterioration is a continuous process from euglycemia to stable hyperglycemia. Simple dichotomous studies may omit crucial factors that contribute to this phenomenon, thus constraining our comprehension and impeding early detection and treatment of glycemic deterioration in DP patients. In this study, we denoted IGT and DEP as the initial and advanced stages of glycemic deterioration, correspondingly, based on the data accessible from DP patients on the platform. Our objective was to examine the changes in gene expression and molecular pathways in DP patients from the euglycemia to the early stages of glycemic deterioration until the onset of diabetes. We combined this information with clinical data to deduce the pathological mechanisms of glycemic deterioration in DP patients, identify promising biomarkers, and validate them through independent cohorts and animal experiments.

## Materials and methods

**Figure 1:**
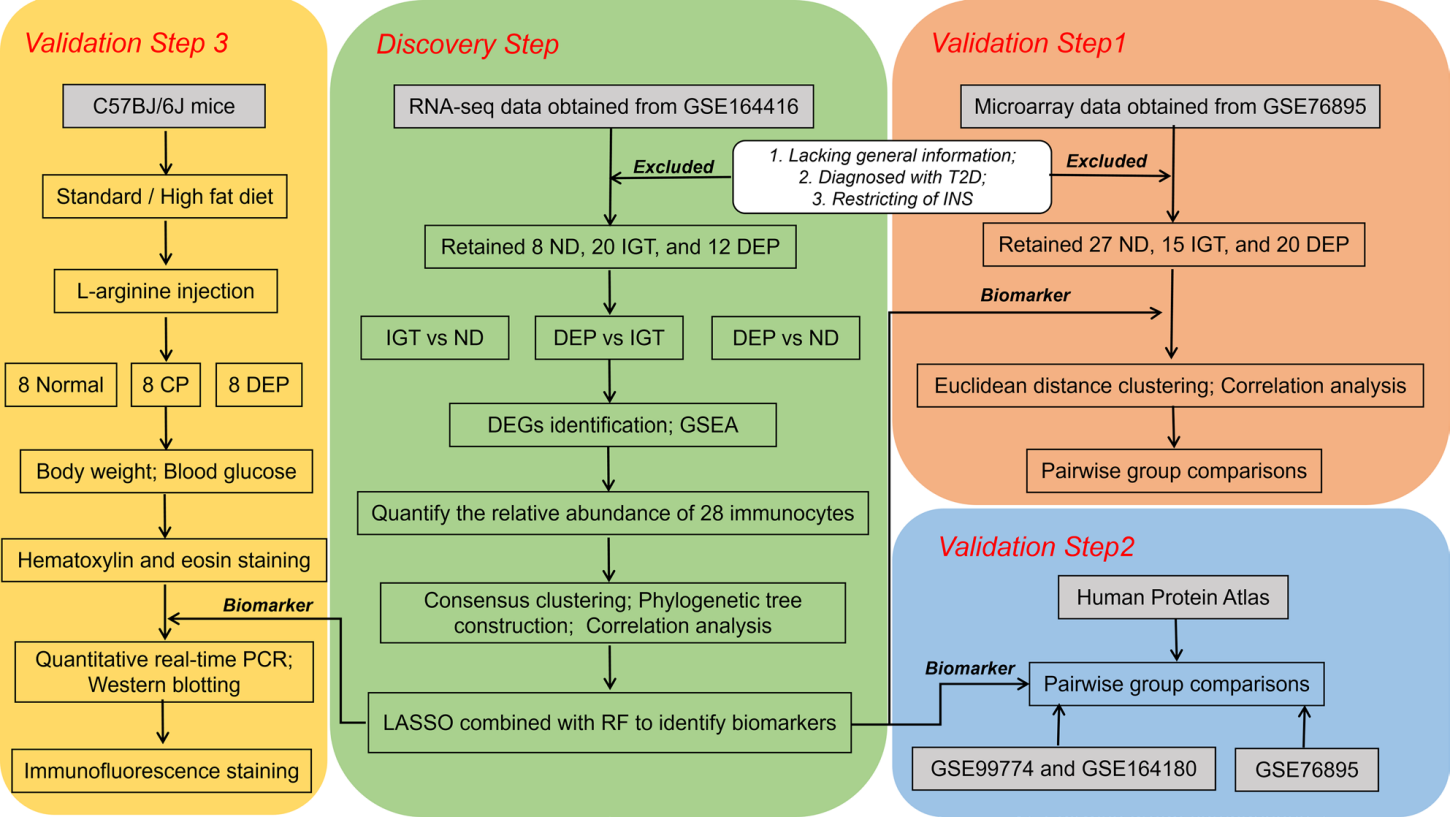
Flowchart of the workflow. Abbreviations are defined as follows: chronic pancreatitis (CP), diabetes of the exocrine pancreas (DEP), differentially expressed genes (DEGs), gene set enrichment analysis (GSEA), impaired glucose tolerance (IGT), least absolute shrinkage and selection operator (LASSO), non-diabetic (ND), random forest (RF), type 2 diabetes (T2D).

This study utilizes data from publicly available databases. Table 1 presents the corresponding datasets, and Fig. 1 depicts the research flow of this study.

**Table 1:** Datasets from publicly available databases in present study.

GEO Accession	Species	Number of samples						
		Normal	Pancreatitis	PA	ND	IGT	T2D	DEP
GSE99774	Mus musculus	8	8	0	0	0	0	0
GSE164180	Mus musculus	0	0	7	0	0	0	0
GSE143754	Homo sapiens	9	6	11	0	0	0	0
GSE164416	Homo sapiens	0	0	0	15	38	39	23
GSE76895	Homo sapiens	0	0	0	27	15	36	20

*DEP* diabetes of the exocrine pancreas, *IGT* impaired glucose tolerance, *PA* pancreatic adenocarcinoma, *ND* non-diabetic, *T2D* type 2 diabetes.

### Data collection and pre-processing

The RNA-seq data (GSE164416) was collected from the Gene Expression Omnibus (GEO) database, and accompanying general information was extracted from the supplementary material by Wigger et al.^19^ in 2021. The expression of marker genes was measured to quantify the probable presence of each patient's cell types, and only patients without disproportionately elevated cell ratios were included. Further information about this process is explained below.

Based on the research by Barovic et al.^8^, we opted to examine the RNA-seq data^19^ acquired from the islets of DP patients. These islets were surgically removed and isolated, allowing us to maintain their pathophysiological features in situ, which was crucial for this study. As outlined by Wigger et al.^19^, all patients underwent surgery to remove pancreatic tumors, pancreatitis, or pancreatic cysts at the University Hospital of TU Dresden. They did not receive chemotherapy before the surgery and did not have any other pancreatic endocrine tumors^19^. The patients were diagnosed based on the fasting glucose, HbA1c, and 2-h glucose thresholds set by the American Diabetes Association (ADA) in the days leading up to surgery^20^. All patients who were pre-diabetic were uniformly classified as IGT^19,20^. For DEP patients, those with a diabetes duration of less than 1 year who tested negative for the presence of circulating autoantibodies against the islets^16,19,21^. In the end, Wigger et al.^19^ included a total of 133 patients, including 18 ND, 41 IGT, 35 DEP and 39 T2D. Patients lacking general information or diagnosed with T2D were excluded from this study. The analysis retained data from 76 patients, comprising 15 ND, 38 IGT, and 23 DEP cases, whose general information was presented in Supplementary Table 1. Raw counts were normalized to transcripts per million (TPM) by gene length in the annotation file (GDC.h38 Flattened GENCODE v22 GFF). The relative percentages of different types of cells in individuals were calculated by the TPM values of marker genes (GCG for alpha cells, INS for beta cells, SST for delta cells, and PPY for gamma cells). In the attempt to at least partly overcome the interference of the study by differences in intra-islets cellular fractions, we restricted the transcriptome analysis to libraries where insulin (INS) is the most expressed gene. Finally, we kept 8 ND, 20 IGT and 12 DEP, and compared the differences in clinical characteristics, including age, body mass index (BMI), HbA1c, and glycaemia at 2-h, across the groups.

### Differentially expressed genes identification and gene set enrichment analysis

Based on the evolution of glycemic deterioration, we produced pairwise group comparisons of IGT versus ND, DEP versus IGT, and DEP versus ND. The raw counts of the pairwise groups were analyzed in DESeq2 (v.1.34.0) and genes with ∣log2FC∣ ≥ 1 and p-value < 0.05 were identified as differentially expressed genes (DEGs) among the groups. The top and bottom 1000 genes were extracted from the list of all genes, which were ordered based on their log2FC between the groups. The gene symbols were then converted to Entrez-ID using org.Hs.eg.db (v.3.14.0). The sorted Entrez-ID was subsequently used as input for clusterProfiler (v.4.2.2)^22^. The gene set enrichment analysis (GSEA) was supported by using the gseGO and gseKEGG functions.

### Immunocyte infiltration analysis

The single sample gene set enrichment analysis (ssGSEA) was conducted to quantify the relative abundance of 28 immunocytes in the islet microenvironment. Marker genes for various immune cells were sourced from the study of Charoentong et al^23^ in 2017. The normalization of the enrichment score was executed to illustrate the relative abundance of different immunocytes, with a bottom and top measurement value of 0 and 1 for each immunocytes, accordingly. The strength of immunocyte infiltration in each patient was calculated by adding up the normalized enrichment scores of 28 immunocytes. To assess the correlation between subpopulations of immunocytes, the Pearson correlation coefficient was employed.

### Consensus clustering and phylogenetic tree construction

A consensus matrix, containing the normalized enrichment scores of 28 immunocytes for each patient, was used for clustering via ConsensusClusterPlus (v.1.58.0)^24^ and constructing a phylogenetic tree by means of ggtree (v.3.2.1)^25^. The t-distributed stochastic neighbour embedding (t-SNE) was run using Rtsne (v.0.15) to determine cluster stability.

### Biomarkers of glycemic deterioration identification

Gene expression was compared separately for the two IGT classes, leading to the identification of DEGs. Additionally, we conducted a sequential analysis utilizing the least absolute shrinkage and selection operator (LASSO) regression and random forest (RF) to identify signature genes that can accurately differentiate between the two classes of IGT. We then used the shared genes as potential biomarkers. We proceeded to examine the expression of these biomarkers amongst groups including ND, T2D and DEP. Finally, we assessed the correlation between biomarkers and clinical characteristics in individuals.

### Biomarkers validation

Biomarkers were validated using microarray data (GSE76895) sourced from the GEO database. It is noteworthy that the patients in GSE76895 shared a common origin with those in the University Hospital of TU Dresden, and thus had similar inclusion criteria and treatment strategies to those in GSE164416. Consequently, our patient selection strategy mirrored that of GSE164416, resulting in the inclusion of 27 ND, 15 IGT, and 20 DEP. Relevant background details can be found in Supplementary Table 2. The affy^26^ package was used to download and read the raw data, which was then normalized via the robust multiarray averaging (RMA) algorithm. Following this, IGT was clustered based on biomarker expression and different IGT classes were identified. Additionally, ND, T2D, and DEP were included for intergroup biomarker comparison, and we similarly analyzed the correlation.

The receiver operating characteristic (ROC) curves were plotted to evaluate the diagnostic efficacy of clinical characteristics including age, BMI, HbA1c, fasting glucose, and glycemia at 2-h, as well as biomarkers in group comparisons. A larger area under the curve indicates a better diagnostic efficacy for the corresponding factor.

Additionally, the expression of biomarkers in the independent cohort was reanalyzed. We acquired expression profiling data (GSE99774, GSE164180, and GSE143754)^27–29^ from the GEO database for both normal individuals and individuals with DP, obtained from patient tissue or animal models. The data went through normalization and correction for batch effects before comparing the groups. Additionally, we examined biomarker immunohistochemical data within pancreatic tissue of normal individuals and those with adenocarcinoma from the Human Protein Atlas (HPA) database^30^.

### Mouse model construction

Twenty-four C57BJ/6J mice (SPF grade, 8 weeks old, male) were obtained from the Experimental Animal Center of Dalian Medical University. After one week of adaptive feeding, the mice were randomly divided into three groups consisting of normal (n=8), CP (n=8), and DEP (n=8). To establish the model, mice in the CP and DEP groups were intraperitoneally injected with L-arginine (350 μg/g/day) for 6 weeks, while the normal group was given the same dose of 0.9% saline. Throughout the study period, the mice in the normal or CP groups were administered a standard diet, whereas the mice in the DEP group were given a diet enriched with high fat content. The body weights of all mice were measured and recorded on a weekly basis. After six weeks, the mice were anaesthetized by intraperitoneal injection of chloral hydrate (250 mg/kg), followed by the collection of blood samples from the tail vein to measure blood glucose. After taking blood samples, the mice were euthanized, and the pancreas was extracted and separated into two sections: (1) preserved at -80℃ for cryopreservation. (2) fixed in a 4% paraformaldehyde solution. The study was authorized and reviewed by the Dalian Medical University Animal Care and Ethics Committee (No: AEE21052).

### Hematoxylin and eosin staining

The fixed pancreatic tissues from mice were dehydrated, embedded in paraffin, and sectioned at 4 μm. The paraffin sections were deparaffinised, followed by staining with haematoxylin and eosin. Ethanol was used for dehydration and xylene was used for clearing. The sections were then sealed with neutral gum and photographed under a light microscope (BX51, Olympus, Japan).

### Quantitative real-time PCR

Total RNA was extracted from the mouse pancreatic tissue using the RNAeasy Animal RNA Extraction Kit (R0024, Beyotime Biotechnology, Shanghai, China). Quantitative real-time quantitative PCR (qRT-PCR) was conducted using the cDNA Synthesis Kit (D7168S, Beyotime Biotechnology, Shanghai, China) and the AceQ qPCR SYBR Green Master Mix (Q131-02, Vazyme, Nanjing, USA), following the manufacturer's protocols. The primers used for qRT-PCR were as follows: B2M (forward, 5′-ACAGTTCCACCCGCCTCACATT-3′; reverse, 3’-TAGAAAGACCAGTCCTTGCTGAAG-5’); GAPDH (forward, 5′-CATCACTGCCACCCAGAAGACTG-3′; reverse, 3’-ATGCCAGTGAGCTTCCCGTTCAG-5’). Standardization of data was performed on GAPDH expression.

### Western blotting

Total protein was extracted from mice pancreatic tissue using a mixture. The protein concentration was determined through usage of the BCA Protein Concentration Assay Kit (P0010, Beyotime Biotechnology, Shanghai) and then the protein samples were transferred to a polyvinylidene difluoride membrane (ISEQ00010, Millipore, USA) before being separated by 10% sodium dodecyl sulphate polyacrylamide gel electrophoresis (S8010, Solarbio, Beijing, China). After blocking, incubation of the primary antibody took place overnight at 4 °C, followed by a 2-h incubation of the secondary antibody at room temperature. Chemiluminescence was performed by utilizing the ECL kit (180-5001, Tanon, Shanghai, China), and subsequently, images were captured. Using ImageJ software, the relative protein levels were calculated. To normalize protein expression levels, Beta-actin was employed.

### Immunofluorescence staining

Following fixation with 4% paraformaldehyde, mouse pancreatic tissue sections were processed for embedding in paraffin, and then deparaffinized utilizing xylene and ethanol. The sections were treated with Citrate buffer (C1010, Solarbio, Beijing, China) which was heated and subsequently immersed. The mixture was then reduced to low heat and left to process for a duration of 15 min. To prevent nonspecific binding, the sections were blocked with a solution containing 3% hydrogen peroxide for a duration of 20 min. Sections were incubated with either insulin or glucagon primary antibodies overnight at 4 °C, then incubated with secondary antibodies for 1 h. Cell nuclei were stained with a DAPI solution, and sections were blocked with an anti-fluorescence quenching sealer (AR1109, Boster, Wuhan, China). Lastly, the sections were captured using an inverted fluorescence microscope (NIB900, Leica Microsystems, Germany).

### Statistics and visualization

Statistical information for each experiment is presented in the Figures, Figure legends, and Figure notes. The violin plot and scatter plot were created and displayed using ggstatsplot (v.0.9.1). The other plots, such as heatmaps and volcano plots, were generated using rstatix (v.0.7.0) and displayed using ggplot2 (v.3.3.5) or ggtree (v.3.2.1)^25^. Shapiro-Wilk and Levene's tests were utilized to establish whether the data adhered to normal distribution and variance chi-square, respectively. In cases where the data met both normal distribution and chi-square, we employed parametric tests (Student’s t-test or Welch’s ANOVA) to assess differences between groups. Conversely, if this was not the case, we utilized non-parametric tests (Wilcoxon test or Kruskal-Wallis test), followed by the Games-Howell test. The Pearson correlation coefficient was employed to examine the relationship between two continuous variables displayed in the scatter plot. Only the initial use of statistical methods or color schemes are annotated, with subsequent occurrences conforming to the first annotation. Statistically significant was determined at *p* < 0.05.

### Ethical approval

GEO is part of public databases, and ethical approval has been obtained for the patients involved. Users can access the relevant data for research purposes and publish appropriate articles. As our study is based on open-source data, there are no ethical concerns or other conflicts of interest.

## Results

### Establishment of the study cohort based on marker genes

**Figure 2:**
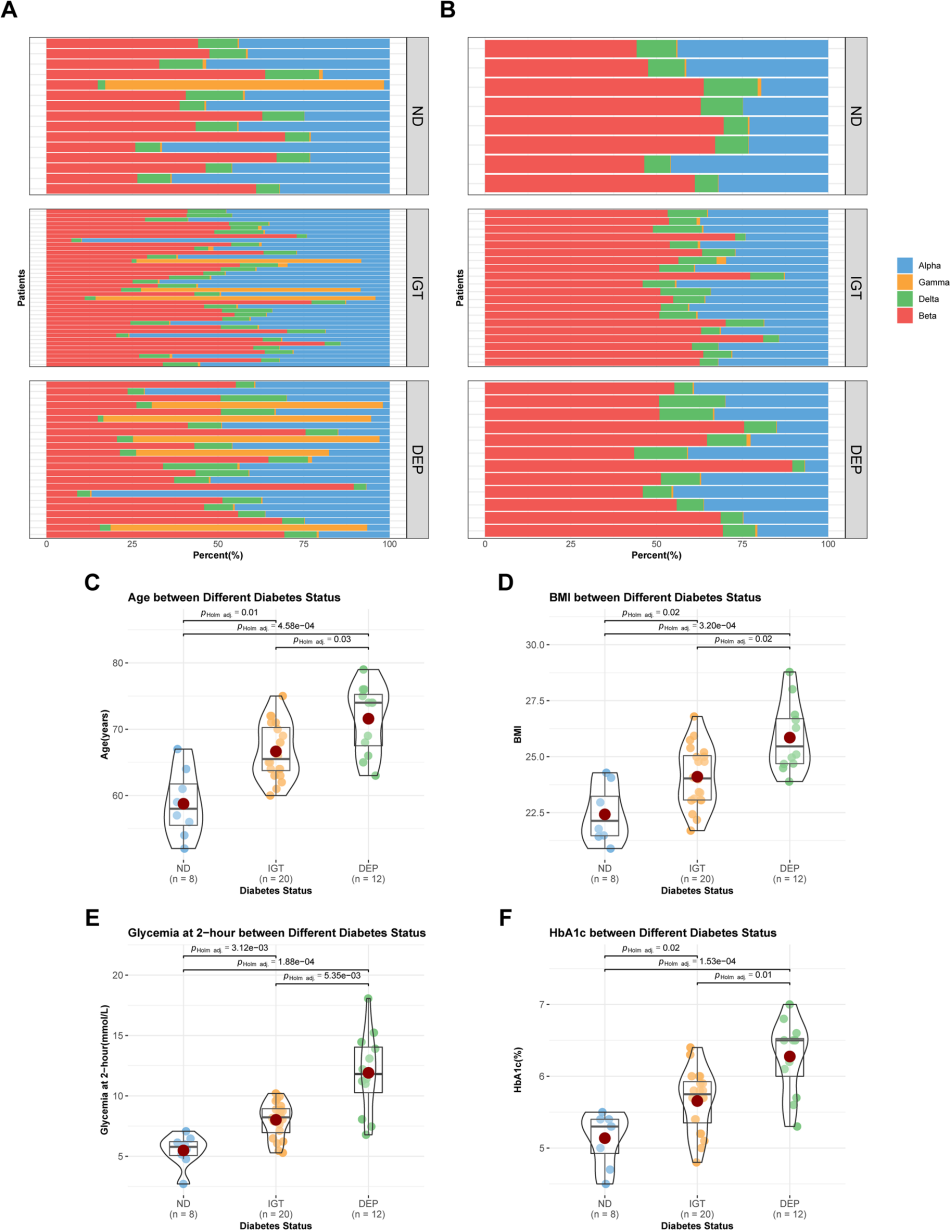
Cell types and clinical characteristics in the study cohort. (**A**) The relative abundance of cells based on marker genes in the original cohort. Alpha cells, blue; Gamma cells, orange; Delta cells, green; Beta cells, red. (**B**) The relative abundance of cells based on marker genes in the restricted cohort. (**C**–**F**) The violin plot of the clinical characteristics of patients. Component comparison was conducted using Welch's one-way ANOVA and Games-Howell test. ND, blue; IGT, orange; DEP, green.

The RNA-seq data and accompanying general information were retrieved from publication, which consisted of islet specimens from 133 adults suffering from either pancreatitis, pancreatic tumors, or pancreatic cysts. Following the removal of patients who did not have matching general information or were diagnosed with T2D (18 and 39, respectively), 15 ND, 38 IGT, and 23 DEP were retained. Following quantification of cell types based on marker genes, beta cells were found to be present in every patient, however, their relative abundance was not always dominant (Fig. 2A). Previous studies^31–33^ have noted that beta cells can convert into gamma cells during pancreatic digestion, leading to changes in the pathophysiological properties of the islets. In view of this, we excluded individuals with abnormally high proportions of gamma cells (Fig. 2B). Additionally, it was discovered that the quantity of alpha cells was elevated in the remaining patients, although less numerous than the beta cell-rich individuals within the same group (Fig. 2A). Limiting the beta cell proportion may aid in detecting additional genes that differed between the groups, as demonstrated in Wigger et al^19^. When attempting to analyze all samples, we discovered that the presence of outlier samples significantly reduced the statistical power of clinical characteristics (Supplementary Figure 1). As a result, we only included individuals rich in beta cells in our study to avoid confounding. Ultimately, our study cohort consisted of 8 ND, 20 IGT, and 12 DEP. The average age of patients in the ND group was 59, which was lower than both the IGT and DEP groups (all *p* < 0.05) (Fig. 2C). The BMI of the three patient groups sequentially increased, with means of 22, 24, and 26, respectively (all *p* < 0.05) (Fig. 2D). Additionally, IGT and DEP groups had significantly higher levels of HbA1c and glycemia at 2-h (all *p* < 0.05) (Fig. 2E,F).

### The dysregulation of gene pathways becomes exacerbated with glycemic deterioration

**Figure 3:**
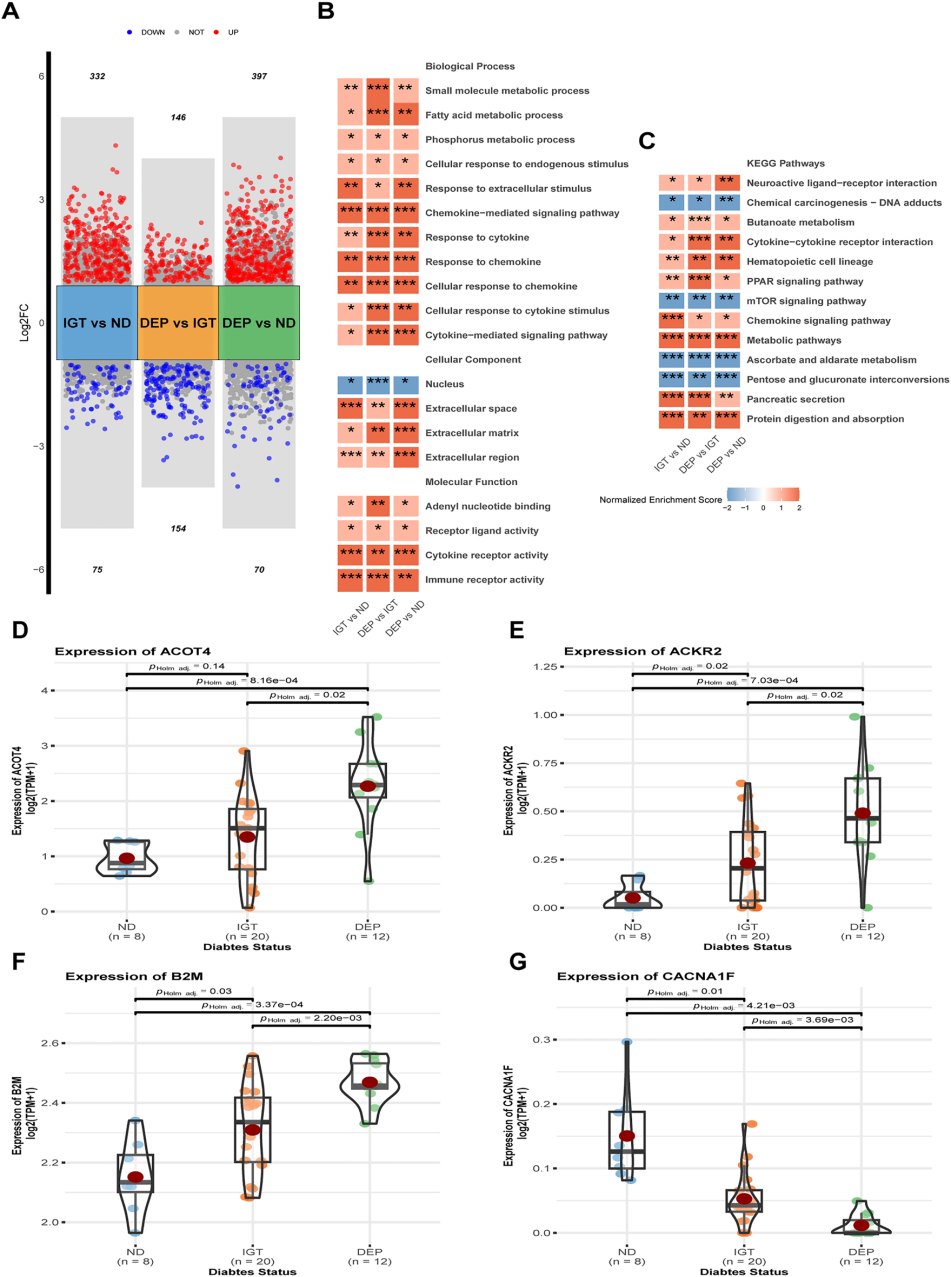
The DEGs and gene pathways across pairwise group comparisons. (**A**) Difference analysis results across pairwise group comparisons. Red, upregulated genes; Blue, downregulated genes; Grey, unregulated genes. (**B**) The Gene Ontology (GO) enrichment results across pairwise group comparisons. The terms were colored using the normalized enrichment score and labeled by the corresponding p-value (*p < 0.05, **p ≤ 0.01, ***p ≤ 0.001). (**C**) The Kyoto Encyclopedia of Genes and Genomes (KEGG) enrichment results across pairwise group comparisons (*p < 0.05, **p ≤ 0.01, ***p ≤ 0.001). (**D**–**G**) The violin plot of the expression of ACOT4, ACKR2, B2M and CACNA1F. Component comparison was conducted using Student’s t-test and Games-Howell test.

Further analysis found varying numbers of DEGs between pairwise group comparisons (Fig. 3A). In particular, there were 407 DEGs in IGT compared to ND (332 upregulated, 75 downregulated), 300 DEGs in DEP compared to IGT (146 upregulated, 154 downregulated), and 467 DEGs in DEP compared to ND (397 upregulated, 70 downregulated). The fold change of 128 DEGs was found to be similar in both IGT versus ND as well as in DEP versus ND. In addition, 45 DEGs were shared between DEP and ND as well as DEP and IGT. However, only 4 DEGs exhibited consistently similar fold changes in all pairwise comparisons (see Supplementary Table 3). Notably, with glycemic deterioration, the expression of Acyl-CoA Thioesterase 4 (ACOT4), Atypical Chemokine Receptor 2 (ACKR2), Beta-2-Microglobulin (B2M), whereas the expression of Calcium Voltage-Gated Channel Subunit Alpha1 F (CACNA1F) was downregulated (Fig. 3D–G). For gene set enrichment analysis, we concentrated on the outcomes of cross evaluations performed in pairwise comparisons between groups. The findings demonstrate significant upregulation of fatty acid metabolism, cellular responses to immune active substances, ligand-receptor activity, and pancreatic secretion (all *p* < 0.05), while the ascorbate and aldarate metabolism, pentose and glucuronate interconversions, and the mTOR signaling pathway were downregulated with glycemic deterioration (all *p* < 0.05) (Fig. 3B,C). Most dysregulated biological processes showed a higher normalized enrichment score in DEP compared to ND or DEP compared to IGT, rather than IGT compared to ND. This indicates that glycemic deterioration leads to an increased biological dysfunction of the islets in individuals.

### Immunocyte infiltration is progressively aggravated with glycemic deterioration

**Figure 4:**
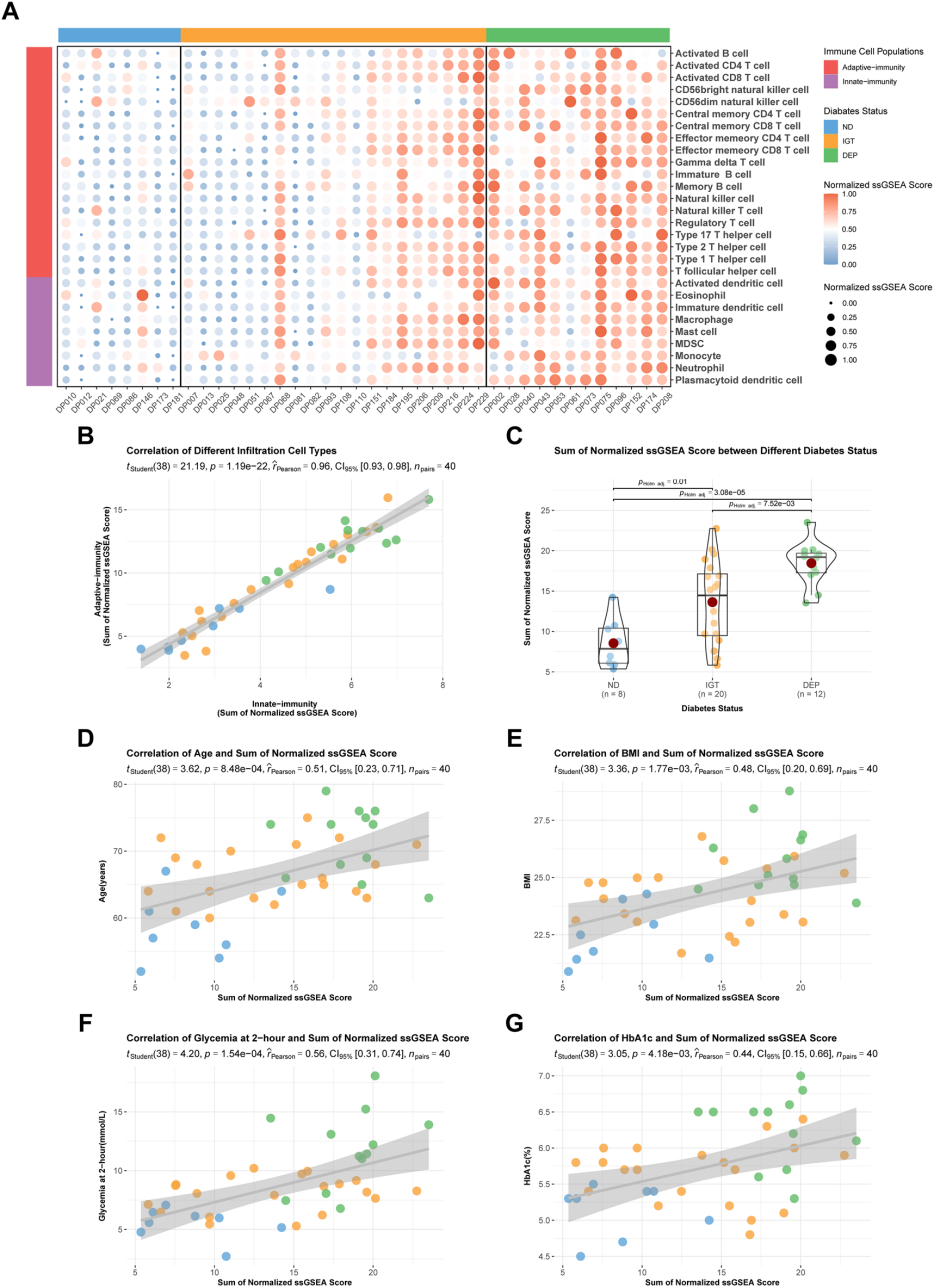
The immunocyte infiltration traits in the restricted cohort. (**A**) A heatmap of the relative abundance of 28 immunocytes in the restricted cohort. The relative abundance of immunocytes was colored and sized according to the normalized score. The sidebars were colored based on diabetes status or immunocyte population (ND, IGT, DEP as above; Innate im-munity, purple; Adaptive immunity, red). (**B**) Correlation of the sum of normalized score between innate immunity and adaptive immunity. Pearson’s correlation coefficient was used to check the association. The grey line is the fitted curve. (**C**) A violin plot of the sum of normalized score between different diabetes status. (**D**–**G**) Correlation between the clinical characteristics and the sum of normalized score.

We have estimated and visualized the relative abundance of 28 immunocytes from ND to DEP (Fig. 4A). Our observations showed that immunocytes were more abundant in DEP than in ND, and the immunocyte infiltration in IGT was not consistent, with some individuals having higher or lower immunocyte infiltration. Further analysis indicated a gradual increase in immunocyte infiltration from ND to DEP (all *p* ≤ 0.01) (Fig. 4C), with positive associations observed between two categories of immunocytes in the local environment (r = 0.96; *p* < 0.01) (Fig. 4B). Additionally, a positive correlation was found between the clinical characteristics and immunocyte infiltration in patients (r = 0.51, 0.48, 0.56, 0.44; all *p* < 0.01) (Fig. 4D–G). Correlation analyses aimed at immunocyte subpopulations revealed a significant positive correlation (all *p* < 0.05) between infiltration abundance of macrophage, natural killer cell, regulatory T cell, type 1 T helper cell and type 2 T helper cell in the pancreatic islet microenvironment of DEP patients (Supplementary Figure 2).

### The heterogeneity of immunocyte infiltration in IGT correlates with glycemic deterioration

**Figure 5:**
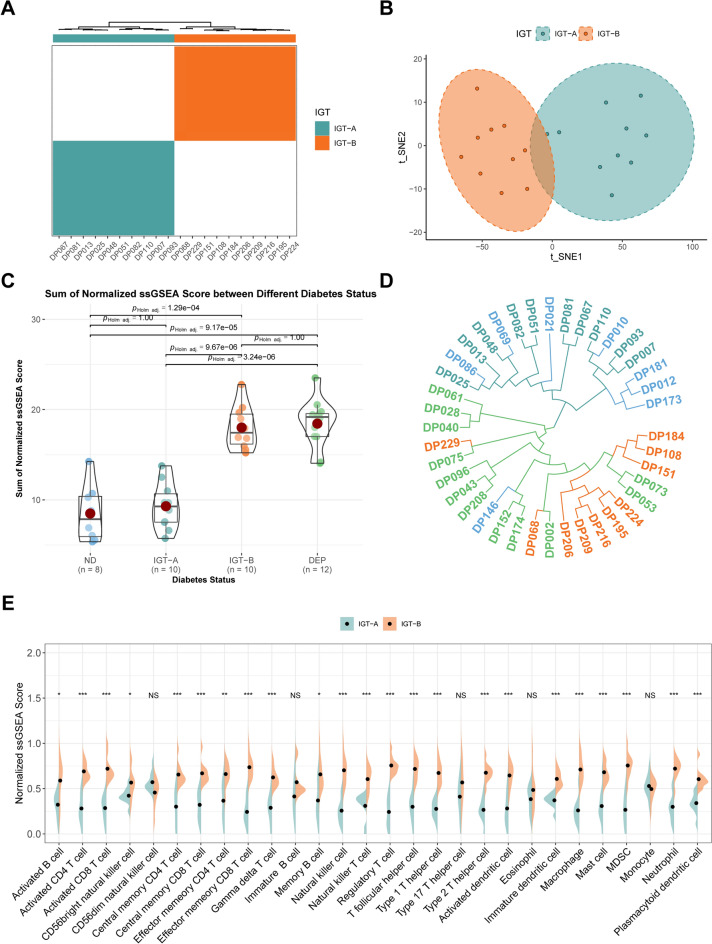
The heterogeneity of immunocyte infiltration in IGT. (**A**) A consensus clustering result within the IGT. The rectangle and top bar were colored according to the IGT cluster (IGT-A, darkcyan; IGT-B, darkorange). (**B**) The t-SNE plot between IGT-A and IGT-B. A scatter represents a unique individual. (**C**) The violin plot of the sum of normalized ssGESA score between different diabetes status. (**D**) The phylogenetic tree was constructed for all patients. (**E**) The split violin plot of the normalized score for different immunocytes between IGT-A and IGT-B. Component comparison using Student’s t-test and Games-Howell test (NS, *p* > 0.05; *p < 0.05; **p ≤ 0.01; ***p ≤ 0.001).

The patients in IGT were categorized based on immunocyte infiltration, resulting in the formation of two clusters: Each cluster contained 10 patients (Fig. 5A). According to t-SNE, patients in IGT-A were grouped together and segregated from those in IGT-B (Fig. 5B). Immunocyte infiltration was found to be more abundant in IGT-B than IGT-A. Differences in immunocyte infiltration were also observed between ND and DEP (all *p* < 0.01). However, no such differences were found between ND and IGT-A, or IGT-B and DEP (all *p* > 0.05) (Fig. 5C). Evaluation of different clinical characteristics between IGT-A and IGT-B did not yield any significant differences (all *p* > 0.05). Similarly, no significant contrasts were noted in some of the clinical characteristics between ND and IGT-A, or IGT-B and DEP (all *p* > 0.05) (Supplementary Figure 3A–D). Furthermore, there was a substantial positive correlation between immunocyte infiltration and clinical characteristics in both IGT-A and IGT-B (r = 0.50, 0.47, 0.56, 0.44; all *p* < 0.01) (Supplementary Figure 3E–H). Following a thorough analysis, ND and IGT-A were grouped together while IGT-B and DEP were placed in a separate branch. Importantly, the two branches were independent from each other (Fig. 5D). We subsequently compared immunocytes infiltration levels between the different branches and found that levels were significantly higher in IGT-B compared to IGT-A. This trend was also observed between ND and DEP (all *p* < 0.05) (Fig. 5E and Supplementary Figure 4A), but not between ND and IGT-A or IGT-B and DEP (all *p* > 0.05) (Supplementary Figure 4B,C).

### B2M, a biomarker of glycemic deterioration in DP patients

**Figure 6:**
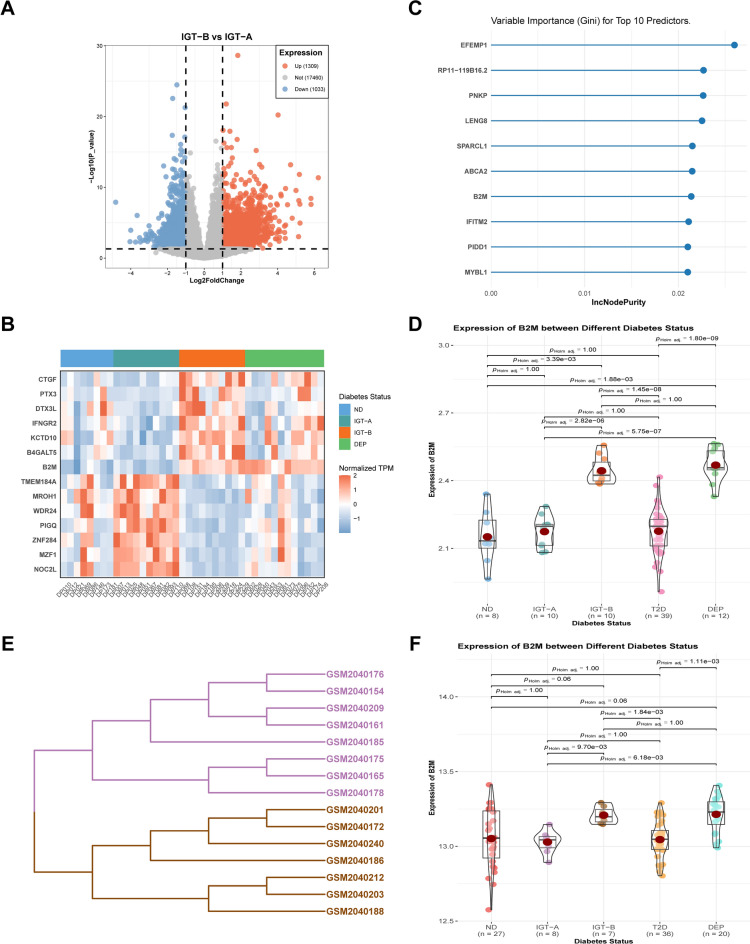
Identification and validation of biomarkers of glycemic deterioration in DP patients. (**A**) The volcano plot of DEGs in IGT-B versus IGT-A. Red, upregulated genes; Blue, downregulated genes; Grey, unregulated genes. (**B**) The normalized TPM value for 14 DEGs that were identified via the LASSO regression. (**C**) The variable importance of top 10 predictors. (**D**) The violin plot of the expression of B2M between different diabetes status in the GSE164416 dataset. (**E**) A Euclidean distance clustering result within the IGT in the GSE76895 dataset. The lines were colored according to the IGT cluster (IGT-A, lightblue; IGT-B, lightyellow; T2D, pink). (**F**) The violin plot of the expression of B2M between different diabetes status in the GSE76895 dataset.

Initially, we identified 2342 DEGs, consisting of 1309 upregulated and 1033 downregulated, in IGT-B compared to IGT-A (Fig. 6A). Subsequently, utilizing the LASSO regression, we classified 14 DEGs with high accuracy in distinguishing between IGT-A and IGT-B. These comprised seven upregulated genes (CTGF, PTX3, DTX3L, IFNGR2, KCTD10, B4GALT5, and B2M) and seven downregulated genes (TMEM184A, MROH1, WDR24, PIGQ, ZNF284, MZF1, and NOC2L) (Fig. 6B). Notably, we observed differential expression of these genes between ND and DEP, and their expression patterns were consistent with IGT-A and IGT-B, respectively. Furthermore, we selected the top 10 genes that discriminated between IGT-A and IGT-B using RF algorithm (Fig. 6C). Upon comparing the two sets of results, only one gene, B2M, was identified as a potential biomarker by both algorithms. Further analysis indicated that the expression of B2M was significantly higher in IGT-B and DEP than in ND and IGT-A (all *p* < 0.01). However, no significant difference was observed between ND and IGT-A or IGT-B and DEP (all *p* > 0.05) (Fig. 6D). Moreover, there was no notable alteration (all *p* > 0.05) in B2M expression in T2D compared to ND or IGT-A, notwithstanding the fact that B2M expression was significantly lower in T2D than in IGT-B or DEP (all *p* < 0.01) (Fig. 6D).

We subsequently validated B2M in the GSE76895 dataset. It is important to note that patients without general information or diagnosed with T2D were removed (5 and 36 respectively), and no patients were excluded due to abnormally high cell ratios (Supplementary Figure 7A). Using the expression of B2M, we performed Euclidean distance clustering in IGT and observed that the patients were separated into two distinct endotypes (see Fig. 6E). We discovered that these corresponded to the two categories of IGT in GSE164416, resulting in them being labelled as IGT-A and IGT-B. Furthermore, the pairwise group comparisons for B2M demonstrated similar findings to those demonstrated previously (Fig. 6F). Significant and positive correlations between the expression of B2M and clinical characteristics were observed in both datasets (r = 0.63, 0.47, 0.26, 0.54, 0.44, 0.39; all *p* < 0.05) (Supplementary Figure 5A–G). The only exception was found in GSE67895, where there was no significant correlation between B2M expression and the age of the patients (r = 0, *p* > 0.05) (Supplementary Figure 5E). The B2M demonstrated a higher area under the ROC curve in several comparisons, such as ND_DEP, IGT-A_IGT-B, ND_IGT-B and IGT-A_DEP, compared to the clinical characteristics (including age, BMI, HbA1c, and glycaemia at 2-h) in the GSE164416 dataset. However, B2M did not exhibit superior diagnostic performance in ND_IGT, ND_IGT-A, and IGT-B_DEP (Supplementary Figure 6A). A similar trend was noticed in the dataset GSE76895 (Supplementary Figure 6B).

Higher expression of B2M was found in pancreatic tissue of patients with chronic pancreatitis (CP) or pancreatic tumors than in normal individuals in the independent cohort (all *p* < 0.05). There was no significant difference between them (*p* > 0.05) (Supplementary Figure 7C). This finding was consistent with the data from pancreatic tissue of animal models (*p* ≤ 0.01) (Supplementary Figure 7B). Additionally, the immunohistochemical results displayed the overexpression of B2M in patients with pancreatic adenocarcinoma (Supplementary Figure 7D).

### B2M validation, based on animal models

**Figure 7:**
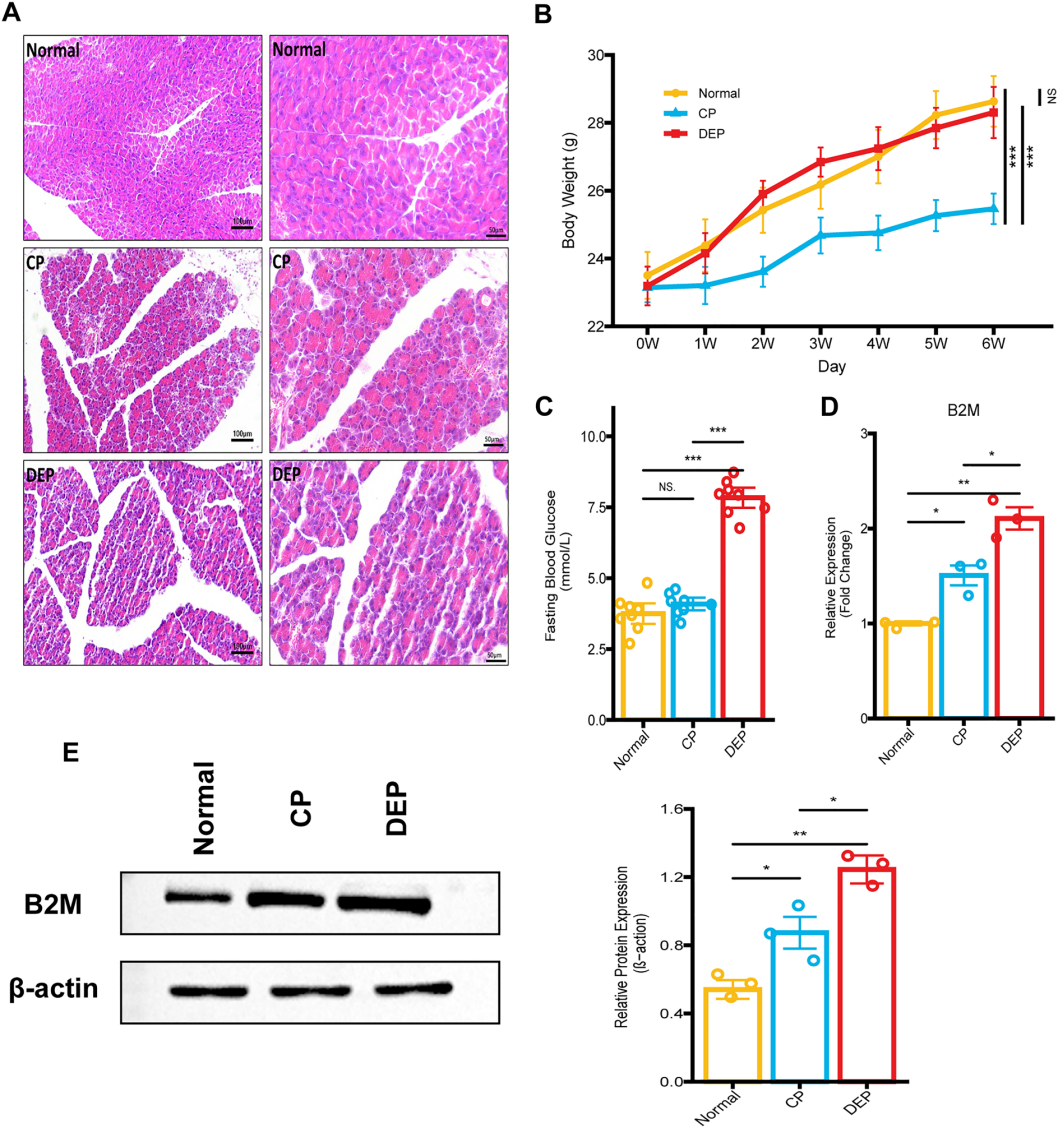
Mouse model construction and B2M expression detection. (**A**) Hematoxylin and eosin staining of mice. (**B**) The body weight curve of mice (Normal, yellow; CP, blue; DEP, red). (**C**) Fasting blood glucose of mice. (**D**) Quantitative real-time PCR results for B2M of pancreatic tissues in mice. (**E**) Western blotting results for B2M of pancreatic tissues in mice.

**Figure 8:**
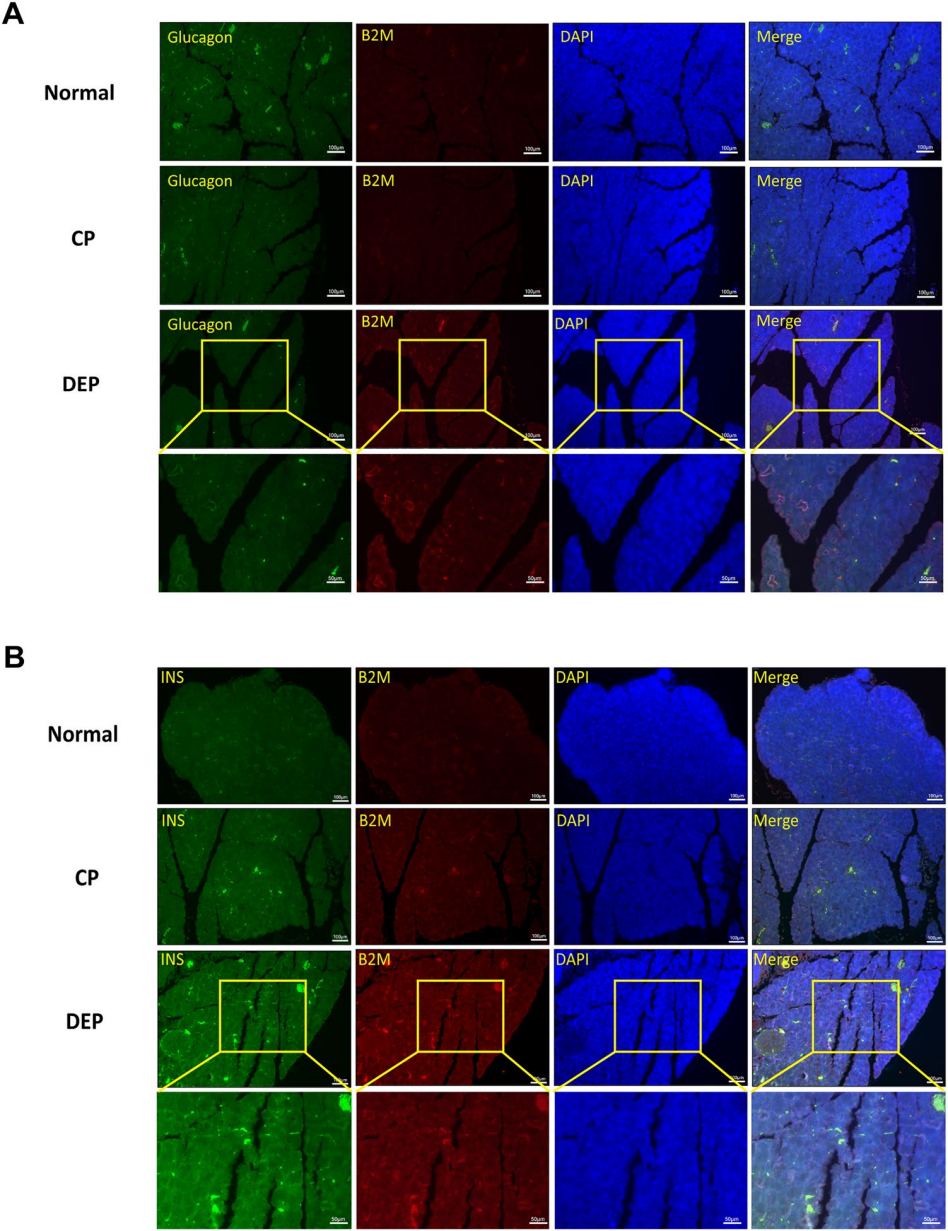
Immunofluorescence staining of pancreatic tissues in mice. (**A**) immunofluorescence for glucagon (green) and B2M (red) in representative samples. (**B**) immunofluorescence for insulin (green) and B2M (red) in representative samples.

As the feeding time was prolonged, there was an increase in the body weights of all mice. At each time point, the body weight of mice in the CP group was notably lower than that of mice in the normal group or the DEP group (all *p* ≤ 0.001). However, there was no significant difference between the normal group and the DEP group (Fig. 7B). Hematoxylin and Eosin (HE) staining showed that the pancreatic tissue of mice in the control group was healthy and free from any signs of bleeding or tissue death, while the islet cells of mice with CP displayed lipid vacuolar degeneration, accompanied by cellular edema and hawks' eye-like megaloblastic lesions in some of the alveoli. The DEP mice suffered from more severe pancreatic tissue destruction than the CP group (Fig. 7A). Fasting blood glucose levels were markedly elevated in DEP mice in comparison to both normal or CP mice. Nevertheless, fasting blood glucose levels did not significantly differ between normal and CP mice (Fig. 7C). Quantitative real-time PCR (qRT-PCR) analysis demonstrated a significant increase in B2M expression in the pancreatic tissues of mice from both CP and DEP groups, as compared to the normal group (all *p* < 0.05). The highest expression was observed in the DEP group (*p* < 0.05) (Fig. 7D). Furthermore, western blotting (WB) of B2M in the pancreatic tissues of mice from all three groups revealed consistent alterations, with notable differences among the groups (all *p* < 0.05) (Fig. 7E). Compared to the normal group, the fluorescence signals of B2M were higher in the pancreatic sections of CP and DEP mice (Fig. 8A,B), with the most abundant signals observed in the DEP group. Within the pancreatic sections of DEP mice, the fluorescence signal of B2M co-localized with glucagon was slightly higher compared to that of CP mice (Fig. 8A), whereas the fluorescence signal of B2M co-localized with insulin was most abundant (Fig. 8B). Meanwhile, the scattered distributions of fluorescence signals of B2M (the location of other endocrine cells or exocrine cells) did not change significantly.

## Discussion

Glycemic deterioration is a common and significant issue among DP patients^34^. Over half of patients with pancreatic tumor are likely to experience IGT or diabetes mellitus as per extensive epidemiological evidence. Patients with pancreatitis are furthermore at a risk of developing de novo diabetes mellitus at a rate of two or more times higher as compared to those without pancreatitis^34^. It is imperative to investigate the pathological mechanisms of glycemic deterioration in DP patients, as this can worsen gastrointestinal and psychiatric burdens, resulting in an increased risk of hospitalization and mortality^7–10^. Early detection and treatment of individual glycemic deterioration is of utmost importance. Previous studies^14,16,19^ have indicated that developing successive patient cohorts can considerably enhance our comprehension of glycemic deterioration in DP patients. As a result, we secured data from three patient groups (ND, IGT, and DEP) from Wigger et al. research^19^. After conducting thorough data preprocessing and quality control measures, we eventually enrolled 40 patients with satisfactory data quality. We examined the clinical features of several groups and discovered a significant discrepancy in age and BMI between patients with IGT or DEP and those with ND, in line with previous population-based studies^1,35–37^. This highlights the dangers of glycemic deterioration in middle-aged and obese DP patients, emphasizing the need for early blood glucose testing among this population. There were overlaps in the DEGs during pairwise group comparisons. Four specific genes: ACOT4, ACKR2, B2M, and CACNA1F exhibited significant differences in any comparison. Amongst these, ACOT4, ACKR2, and B2M demonstrated upregulated expression, whereas CACNA1F exhibited downregulated expression. Additionally, the signal pathways, including those related to fatty acid metabolism, cellular reactions to immune-active substances, and ligand-receptor activity, significantly were enriched with glycemic deterioration. As an important coenzyme for the fatty acid beta-oxidation, ACOT4 has the ability to cleave acyl-CoA thioester in mitochondria, preventing an excessive amount of acyl-CoA thioester from hindering beta-oxidase activity. The upregulation of ACOT4 promotes the fatty acid beta-oxidation, resulting in the generation of more reactive oxygen species (ROS) through the electron transport chain. A distinctive feature of beta cells is their restricted expression of antioxidant enzymes. The upregulation of ACOT4 results in an increase in ROS that heightens the susceptibility of beta cells to oxidative damage, posing a substantial risk for beta-cell dysfunction. On the other hand, ROS efflux from mitochondria to cytoplasm results in the production of inflammatory signaling factors, generating inflammatory signaling factors that lead to immune infiltration and tissue damage. As a constituent of major histocompatibility complex class I (MHC-I), B2M can bind to surface-expressed receptors on cells, leading to the activation of NF-κB and the initiation of local inflammation. Furthermore, it is responsible for stimulating macrophages in the tissue microenvironment, thereby inducing the secretion of pro-inflammatory cytokines^38^. Upon further analysis, it was determined that immunocyte infiltration gradually increased with glycemic deterioration. In patients with DP, inflammation is a significant characteristic, and any pancreas that has suffered damage will trigger an inflammatory reaction. While tissue damage and the ensuing inflammatory response wane in some individuals, but acute inflammation is intensified or persistent low-grade inflammation emerges in most cases^13,39^. At this stage, inflammatory processes stimulate and attract immunocytes through secreted cytokines and chemokines, which in turn secrete cytokines to promote inflammation and cause harm to tissues. Research^40–42^ indicates that the inflammatory environment triggers the secretion of cytokines such as IFN-γ, TNF-α and IL-1β by T cells and macrophages, which further hinder glucose-stimulated insulin release and lead to dysfunction of beta-cells. Meanwhile, a recent study^43^ of patients with acute pancreatitis confirmed that serum levels of IL-1β and IFN-γ are important predictors of individual glycemic deterioration. Additionally, infiltrating immunocyte activate trypsinogen and lead to the demise of follicular cells, whereas low-level inflammation promotes stellate cell activation that induces pancreatic fibrosis. These changes exacerbate the inflammatory response and tissue damage in the microenvironment. The current study revealed a noteworthy, significantly correlation between the degree of immunocyte infiltration, including macrophages, NK cells and regulatory T cells within the islet microenvironment of DEP patients. It has been observed that macrophages and NK cells work together in the inflammatory microenvironment to activate regulatory T cells by presenting antigens, and the activated regulatory T cells regulate their functions in turn^44,45^. This intercellular communication network plays a vital role in promoting inflammatory responses and tissue damage. We hypothesize that the persistent inflammatory response in the exocrine zone of the pancreas, along with the increased expression of ACOT4 and B2M in pancreatic islet tissues of DP patients, exacerbates inflammatory injury by inducing immunocyte infiltration. This contributes to beta-cell dysfunction which disrupts glycemic homeostasis in these patients. Furthermore, a favorable correlation was discovered between the immunocyte infiltration and patients' age, BMI, HbA1c, and glycemia at 2-h. This discovery provides additional evidence that immunocyte infiltration may contribute to glycemic deterioration.

Significantly, IGT exhibited notable heterogeneity in its immunocyte infiltration. Individuals with enhanced immunocyte infiltration had comparable characteristics with DEP in terms of immune cell abundance and clinical features. Meanwhile, those with lower immunocyte infiltration exhibited similarities with ND. It is worth noting that this heterogeneity has been previously reported. Based on unsupervised machine learning identifying 31 expression profiles for cytokines and chemokines, Kimita et al.^46^ displayed the existence of two endotypes that are completely different following an attack of pancreatitis, "inflammatory" and "non-inflammatory". The inflammatory endotype exhibited notably higher expression of 16 cytokines and chemokines in comparison to the non-inflammatory endotype. There were significant changes in pancreatic and intestinal hormones, particularly a marked elevation in the gastric inhibitory peptide (GIP), which is believed to have a significant contribution to post-pancreatitis diabetes (a specific type of DEP). In this study, there were differences observed in the immunocyte infiltration between IGT-A and IGT-B. More significantly, both studies revealed no statistically significant differences in patients' clinical characteristics, such as age, BMI, HbA1c, and glycemia at the 2-h mark, between the various endotypes. Therefore, individuals with the same phenotype may exhibit entirely distinct intrinsic alterations, and such variations are typically linked with particular disease progression. It is crucial to acknowledge that DP patients experience glycemic deterioration due to the gradual increase of immunocyte infiltration within the islet microenvironment. Correspondingly, subjects with a more robust immunocyte infiltration are at an elevated risk of developing DEP. The level of infiltration by 23 immunocytes was markedly higher in IGT-B in comparison to IGT-A. Conversely, there was a significant difference between ND and DEP regarding all 28 immunocytes. However, there were only a few statistically significant differences between ND and either IGT-A or IGT-B and DEP. It is worth noting that higher levels of NK cells infiltrated the group with poorer glycemic status in pairwise group comparisons. These findings are noteworthy as NK cell activity relies on the coordination of activating and inhibitory receptor-ligand interactions, serving as a bridge between innate and adaptive immunity^47,48^. In this study, during the process of glycemic deterioration, the patients displayed significant ligand-receptor activity, and the relative abundance of NK cells also increased gradually. Previous studies have shown that the activation of the natural cytotoxicity receptor NKp46 on the surface of NK cells contributes to the progression of type 1 diabetes, while a notable increase in NK cells in obese patients causes insulin resistance^49,50^. In the past decade, progress has been made in NK-based immunotherapy for treating malignant tumors. It is anticipated that this therapy may aid in glycemic control for patients with DP and future DEP treatment^47,51^.

Glycemia at 2-h and HbA1c are frequently used in clinical practice to assess an individual's short-term or long-term glycemic control. However, glycemic deterioration often occurs following damage to the islets, resulting in the deterioration of these vital organs. In their research, Danielle et al.^52^ utilized commonly-used experimental parameters and biochemical markers to evaluate risk factors for glycemic deterioration in DP patients and created a prediabetes self-assessment screening score. Other studies^53^ have demonstrated the significance of this approach in mitigating the effects of DEP. Nevertheless, its limitations persist due to hysteresis. In order to minimize the limitations of existing assessment protocols as much as possible, we analyzed IGT-A and IGT-B, both of which share the same clinical phenotype but differ significantly in terms of their levels of islet damage. B2M exhibited significant overexpression in IGT-B. Both the LASSO regression algorithm and the random forest algorithm identified B2M as a signature gene that can effectively differentiate IGT-A from IGT-B. L1 penalties were employed by the Lasso regression for feature selection to avoid overfitting and enhance model generalization, which enabled the identification of the most influential candidates from thousands of markers^54^. Random forests outperformed single decision trees in handling large-scale, high-dimensional data and providing precise classification and regression predictions^55,56^. Both algorithms are frequently used to identify biomarkers through gene expression profiles^57–60^. In this study, we examine the genes which have been recognized as feature genes by both algorithms. Limiting the number of markers, we ensure high accuracy. In several datasets, B2M demonstrated higher expression in DEP when compared to ND. Conversely, no significant difference was found in B2M expression between ND and IGT-A or IGT-B and DEP. Following analysis with T2D patients, it was observed that B2M expression in T2D patients was similar to that of ND or IGT-A but significantly lower than that of IGT-B or DEP patients. This indicates that glycemic deterioration in normal individuals may not have a strong association with B2M, and also shows that B2M has the potential to differentiate between T2D and DEP. Therefore, in DP patients (particularly those who already exhibit changes in blood glucose levels), the extent of islet destruction may surpass our presumptions, even if the patient's current clinical features are insufficient to diagnose with DEP. Meanwhile, individuals with uniform phenotypes but comparatively severe islet destruction demonstrated elevated expression of B2M and had a greater likelihood of developing DEP.

Animal experiments revealed that B2M transcript and protein levels were notably higher in the pancreatic tissues of DEP mice than in CP mice, further confirming B2M's role in the glycemic deterioration. Immunofluorescence analysis showed increased fluorescent signals of B2M co-localized with alpha cells (glucagon-positive) and beta cells (insulin-positive) in pancreatic sections from DEP mice compared to CP mice. Notably, the fluorescent signals co-localized with B2M with beta cells were the most abundant. The distribution of B2M fluorescent signals in the remaining regions (the location of other endocrine and exocrine cells) was less abundant and did not differ significantly between groups. These results confirm the up-regulation of B2M expression during the glycemic deterioration in DP, affirming the predictive utility of B2M as a biomarker and suggesting that elevated B2M is closely associated with beta cells and alpha cells. Biomarkers are utilized to distinguish between the interest diseases or states. Transcriptomics-based biomarkers have been extensively used in the clinic to predict outcomes^61–63^. This study analyzed transcriptomics of living islets from DP patients. The findings suggest that abnormally elevated B2M can be used as a biomarker of glycemic deterioration in DP patients and verified its accurate predictive properties. Additionally, B2M expression was found to be concentrated in alpha and beta cells, as shown by immunofluorescence staining of mouse pancreatic sections. However, it is important to note that the pancreas has a low percentage of islet cells and that B2M is closely linked to inflammation^38,64^. Therefore, Western blotting experiments of mouse whole pancreatic lysates may be affected by the up-regulation of B2M induced by the inflammatory response in other cells of the pancreas, particularly in the exocrine pancreas. This issue requires further exploration using other samples and techniques. In conclusion, B2M can serve as an initial check for glycemic deterioration in DP patients. Early identification and management of aberrant B2M expression are essential for improving glycemic control in DP patients. Physicians could assess the patient's glycemic deterioration risk by detecting B2M expression, and subsequently institute further diagnostic and intervention measures. Furthermore, B2M is pivotal in evaluating DP patients' prognosis. By tracking changes in B2M levels before and after treatment, healthcare professionals can determine the treatment's effectiveness. This data can aid in regulating the care plan and generating individualized treatment, thereby easing the gastrointestinal and psychological burden in DP patients. Moreover, B2M exhibits promise for therapeutic application. By hindering or disrupting the expression or function of B2M, it is projected to boost the quality of life and survival of DP patients.

Whilst our study offers novel biological understandings into the glycemic deterioration among DP patients, certain constraints persist. Primarily, the partial and prejudiced patient data within public records restricted our capacity to recognize and regulate confounding factors, and subsequently led to a reduced sample size included in the study. While the results were verified in subsequent independent datasets and animal trials, more extensive and multicentre cohorts are required to test their extrapolation further. Additionally, due to the restrictions of our current data, we could solely quantify cells using mRNA expressions. This method inevitably neglects the biological processes that may explain changes in mRNA expression. Furthermore, the glycemic deterioration in DP patients involves long-term development, and typically, DEP patients are older than normoglycemic individuals, with an increased risk of glycemic deterioration due to ageing^36^. Therefore, it is not wholly possible to disregard the effect of age variations among the different groups in this study on the current findings. Furthermore, in this study, the type of disease in patients with DP could not be verified due to the absence of a definite diagnosis. Although outlier patients were removed through data preprocessing and quality control, heterogeneity among DP (such as pancreatitis or pancreatic tumors) was not entirely eliminated, which has an impact on our current results^34,65^. Nevertheless, these limitations are common and inevitable in studies that rely on public data, and they do not affect the conclusions or implications of our study.

## Conclusions

Our study found that biological processes such as nutrient metabolism and complex immune response within the islets were disrupted in DP patients with glycemic deterioration. Specifically, there was a progressive increase in immunocyte infiltration in the islet microenvironment, which contributed to glycemic deterioration. In addition, phenotypically consistent individuals with IGT were categorized into two endotypes, IGT-A and IGT-B, with IGT-B exhibiting a greater immunocyte infiltration and having a greater propensity to develop DEP. Crucially, the validated biomarker B2M demonstrated a significant and positive correlation with the immunocyte infiltration and clinical characteristics. As such, it could potentially serve as a biomarker for glycemic deterioration in DP patients.

### Supplementary Information

Supplementary Figure 1.

Supplementary Figure 2.

Supplementary Figure 3.

Supplementary Figure 4.

Supplementary Figure 5.

Supplementary Figure 6.

Supplementary Figure 7.

Supplementary Table 1.

Supplementary Table 2.

Supplementary Table 3.

Supplementary Legends.

Supplementary Information 1.

## Data Availability

The public data used here are available in the GEO database (accession number: GSE164416, GSE76895, GSE99774, GSE164180, and GSE143754; https://www.ncbi.nlm.nih.gov/geo/). The codes can be obtained from the first author upon reasonable request.

## Abbreviations

ADA: American Diabetes Association
AP: Acute pancreatitis
BMI: Body mass index
CP: Chronic pancreatitis
DEGs: Differentially expressed genes
DEP: Diabetes of the exocrine pancreas
DP: Diseases of the exocrine pancreas
GEO: Gene expression omnibus
GIP: Gastric inhibitory peptide
GO: Gene ontology
GSEA: Gene set enrichment analysis
HPA: Human protein atlas
INS: Insulin
IGT: Impaired glucose tolerance
KEGG: Kyoto encyclopedia of genes and genomes
LASSO: Least absolute shrinkage and selection operator
MHC-I: Major histocompatibility complex class I
ND: Non-diabetic
NK: Natural killer
qRT-PCR: Quantitative real-time PCR
RF: Random forest
ROC: Receiver operating characteristic
RMA: Robust multiarray averaging
ssGSEA: Single sample gene set enrichment analysis
T2D: Type 2 diabetes
TPM: Transcripts per million
t-SNE: T-distributed stochastic neighbor embedding
WB: Western blotting
