# Supplementary material for: Mechanism exploration and biomarker identification of glycemic deterioration in patients with diseases of the exocrine pancreas
Source: Sci Rep. 2024 Feb 22;14:4374. doi: 10.1038/s41598-024-52956-x (PMC10883946; doi:10.1038/s41598-024-52956-x)
Supplement: Supplementary file 9 — Supplementary Table 2. [file 41598_2024_52956_MOESM9_ESM.docx]

**Supplementary Table 2.** Baseline clinical characteristic of patients in the GSE76895 dataset.


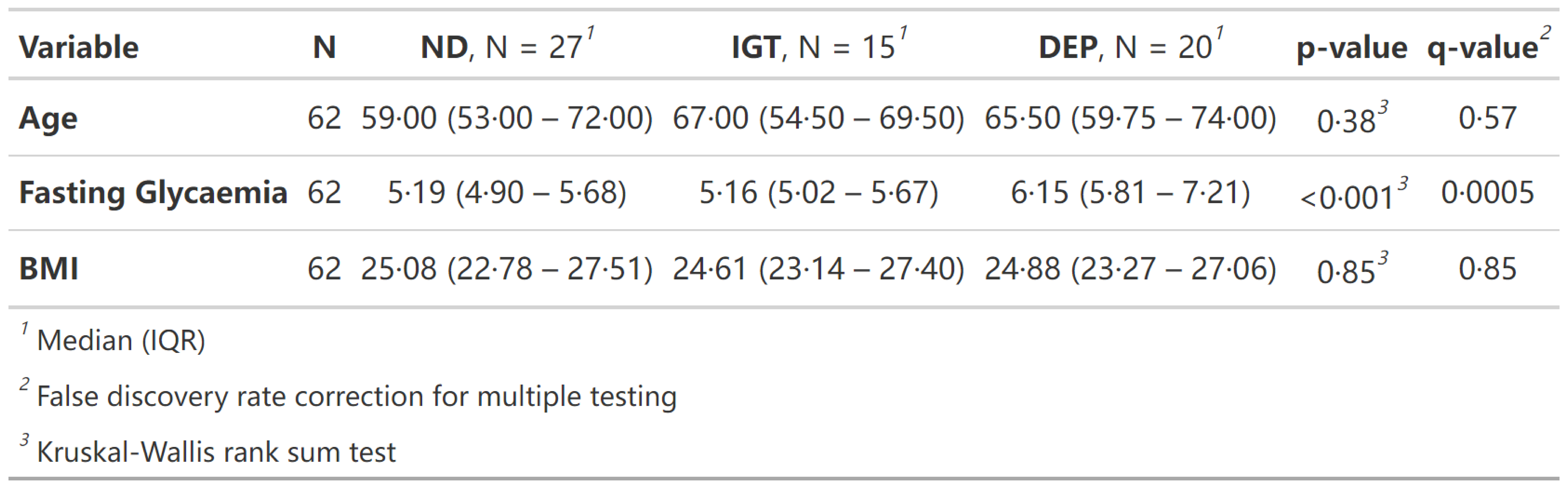


Abbreviations are defined as follows: body mass index (BMI), not have diabetes (ND), impaired glucose tolerance (IGT), diabetes of the exocrine pancreas (DEP).
