# Supplementary material for: Mechanism exploration and biomarker identification of glycemic deterioration in patients with diseases of the exocrine pancreas
Source: Sci Rep. 2024 Feb 22;14:4374. doi: 10.1038/s41598-024-52956-x (PMC10883946; doi:10.1038/s41598-024-52956-x)
Supplement: Supplementary file 10 — Supplementary Table 3. [file 41598_2024_52956_MOESM10_ESM.docx]

**Supplementary Table 3.** Difference analysis results across pairwise group comparisons.

| **Gene Symbol** | **Log2FoldChange** | **P value** | **Regulated** | **Comparison** |
| --- | --- | --- | --- | --- |
| C21orf33 | 4.3131 | 0.0003 | up-regulated | IGT vs ND |
| MEP1B | 3.7303 | 0.0001 | up-regulated | IGT vs ND |
| IL12RB2 | 3.6635 | 0.0076 | up-regulated | IGT vs ND |
| FGA | 3.2868 | 0.0068 | up-regulated | IGT vs ND |
| PDCD1LG2 | 3.2398 | 0.0087 | up-regulated | IGT vs ND |
| SIGLEC8 | 3.1554 | 0.0195 | up-regulated | IGT vs ND |
| NFE2 | 3.1415 | 0.0080 | up-regulated | IGT vs ND |
| C10orf111 | 3.0906 | 0.0007 | up-regulated | IGT vs ND |
| RAB44 | 3.0868 | 0.0106 | up-regulated | IGT vs ND |
| RP11-56B16.5 | 3.0807 | 0.0051 | up-regulated | IGT vs ND |
| CTB-32O4.2 | 3.0561 | 0.0016 | up-regulated | IGT vs ND |
| LAX1 | 3.0492 | 0.0035 | up-regulated | IGT vs ND |
| RP11-270M14.4 | 3.0346 | 0.0091 | up-regulated | IGT vs ND |
| RP4-536B24.3 | 2.9858 | 0.0110 | up-regulated | IGT vs ND |
| CTD-3116E22.7 | 2.9813 | 0.0005 | up-regulated | IGT vs ND |
| SH3TC2 | 2.9752 | 0.0232 | up-regulated | IGT vs ND |
| RP11-336A10.5 | 2.9607 | 0.0052 | up-regulated | IGT vs ND |
| RP11-651P23.5 | 2.9402 | 0.0004 | up-regulated | IGT vs ND |
| ERVFRD-1 | 2.9291 | 0.0222 | up-regulated | IGT vs ND |
| RP11-524F11.1 | 2.8921 | 0.0081 | up-regulated | IGT vs ND |
| LINC01301 | 2.8878 | 0.0065 | up-regulated | IGT vs ND |
| MEIG1 | 2.8362 | 0.0000 | up-regulated | IGT vs ND |
| RP11-28G8.1 | 2.7998 | 0.0194 | up-regulated | IGT vs ND |
| HSPB7 | 2.7761 | 0.0200 | up-regulated | IGT vs ND |
| CEACAM7 | 2.7730 | 0.0048 | up-regulated | IGT vs ND |
| IL4I1 | 2.7683 | 0.0088 | up-regulated | IGT vs ND |
| AC073133.1 | 2.7602 | 0.0056 | up-regulated | IGT vs ND |
| IL2RB | 2.7380 | 0.0073 | up-regulated | IGT vs ND |
| NR1I2 | 2.7257 | 0.0138 | up-regulated | IGT vs ND |
| AC007099.1 | 2.7220 | 0.0163 | up-regulated | IGT vs ND |
| C8orf31 | 2.6944 | 0.0010 | up-regulated | IGT vs ND |
| GIMAP5 | 2.6769 | 0.0045 | up-regulated | IGT vs ND |
| CHRNB4 | 2.6338 | 0.0107 | up-regulated | IGT vs ND |
| MKI67 | 2.6068 | 0.0229 | up-regulated | IGT vs ND |
| RP4-794I6.4 | 2.5759 | 0.0203 | up-regulated | IGT vs ND |
| AC092614.2 | 2.5749 | 0.0120 | up-regulated | IGT vs ND |
| LINC00901 | 2.5571 | 0.0302 | up-regulated | IGT vs ND |
| AC093110.3 | 2.5538 | 0.0136 | up-regulated | IGT vs ND |
| NCCRP1 | 2.5510 | 0.0195 | up-regulated | IGT vs ND |
| TAC4 | 2.5467 | 0.0189 | up-regulated | IGT vs ND |
| ITIH3 | 2.5431 | 0.0018 | up-regulated | IGT vs ND |
| MYO1G | 2.5183 | 0.0183 | up-regulated | IGT vs ND |
| LDB3 | 2.5059 | 0.0005 | up-regulated | IGT vs ND |
| PGF | 2.4801 | 0.0046 | up-regulated | IGT vs ND |
| SIGLEC14 | 2.4741 | 0.0285 | up-regulated | IGT vs ND |
| HCAR1 | 2.4621 | 0.0228 | up-regulated | IGT vs ND |
| BUB1 | 2.4594 | 0.0033 | up-regulated | IGT vs ND |
| APLNR | 2.4507 | 0.0047 | up-regulated | IGT vs ND |
| TNN | 2.4413 | 0.0311 | up-regulated | IGT vs ND |
| CECR7 | 2.4380 | 0.0129 | up-regulated | IGT vs ND |
| C16orf71 | 2.4307 | 0.0012 | up-regulated | IGT vs ND |
| HSF2BP | 2.4099 | 0.0366 | up-regulated | IGT vs ND |
| RP1-149A16.3 | 2.4069 | 0.0228 | up-regulated | IGT vs ND |
| RP5-908M14.10 | 2.4014 | 0.0373 | up-regulated | IGT vs ND |
| KIAA1045 | 2.3995 | 0.0359 | up-regulated | IGT vs ND |
| RP13-349O20.2 | 2.3880 | 0.0176 | up-regulated | IGT vs ND |
| LINC01446 | 2.3836 | 0.0368 | up-regulated | IGT vs ND |
| PMP2 | 2.3661 | 0.0413 | up-regulated | IGT vs ND |
| RP11-379B8.1 | 2.3565 | 0.0391 | up-regulated | IGT vs ND |
| RP11-521C20.5 | 2.3518 | 0.0311 | up-regulated | IGT vs ND |
| RP11-327O17.2 | 2.3492 | 0.0129 | up-regulated | IGT vs ND |
| DAO | 2.3435 | 0.0153 | up-regulated | IGT vs ND |
| NHLH1 | 2.3420 | 0.0331 | up-regulated | IGT vs ND |
| CENPF | 2.3356 | 0.0006 | up-regulated | IGT vs ND |
| RP11-204L24.2 | 2.3249 | 0.0433 | up-regulated | IGT vs ND |
| ZNF730 | 2.3234 | 0.0490 | up-regulated | IGT vs ND |
| HMCN2 | 2.3150 | 0.0410 | up-regulated | IGT vs ND |
| LLNLR-246C6.1 | 2.3132 | 0.0008 | up-regulated | IGT vs ND |
| FAM209A | 2.3032 | 0.0202 | up-regulated | IGT vs ND |
| AC073333.8 | 2.3022 | 0.0342 | up-regulated | IGT vs ND |
| LRRN4CL | 2.3018 | 0.0109 | up-regulated | IGT vs ND |
| RP11-505K9.4 | 2.2910 | 0.0378 | up-regulated | IGT vs ND |
| TREM1 | 2.2644 | 0.0225 | up-regulated | IGT vs ND |
| ASIC4 | 2.2539 | 0.0239 | up-regulated | IGT vs ND |
| C17orf53 | 2.2447 | 0.0204 | up-regulated | IGT vs ND |
| RP11-53B2.1 | 2.2354 | 0.0171 | up-regulated | IGT vs ND |
| RP11-724N1.1 | 2.2314 | 0.0139 | up-regulated | IGT vs ND |
| CTC-265N9.1 | 2.2310 | 0.0143 | up-regulated | IGT vs ND |
| B2M | 2.2277 | 0.0232 | up-regulated | IGT vs ND |
| HOXB-AS1 | 2.2277 | 0.0035 | up-regulated | IGT vs ND |
| LINC01141 | 2.2250 | 0.0115 | up-regulated | IGT vs ND |
| LINC01291 | 2.2067 | 0.0028 | up-regulated | IGT vs ND |
| RP1-187B23.1 | 2.1944 | 0.0119 | up-regulated | IGT vs ND |
| RP11-276E15.4 | 2.1840 | 0.0109 | up-regulated | IGT vs ND |
| OASL | 2.1839 | 0.0369 | up-regulated | IGT vs ND |
| PACRG-AS1 | 2.1685 | 0.0220 | up-regulated | IGT vs ND |
| SPAG5-AS1 | 2.1670 | 0.0034 | up-regulated | IGT vs ND |
| PKD1L1 | 2.1647 | 0.0170 | up-regulated | IGT vs ND |
| SPATA25 | 2.1550 | 0.0221 | up-regulated | IGT vs ND |
| TRPC7 | 2.1528 | 0.0378 | up-regulated | IGT vs ND |
| ITGAL | 2.1453 | 0.0061 | up-regulated | IGT vs ND |
| NUDT8 | 2.1421 | 0.0081 | up-regulated | IGT vs ND |
| RP11-554D20.2 | 2.1412 | 0.0207 | up-regulated | IGT vs ND |
| AHNAK2 | 2.1412 | 0.0011 | up-regulated | IGT vs ND |
| RP11-374A4.1 | 2.1366 | 0.0132 | up-regulated | IGT vs ND |
| CTD-3032J10.4 | 2.1238 | 0.0382 | up-regulated | IGT vs ND |
| MME | 2.1180 | 0.0480 | up-regulated | IGT vs ND |
| LINC01482 | 2.1172 | 0.0233 | up-regulated | IGT vs ND |
| GRIP2 | 2.0926 | 0.0269 | up-regulated | IGT vs ND |
| RGS8 | 2.0904 | 0.0110 | up-regulated | IGT vs ND |
| AC004156.3 | 2.0896 | 0.0336 | up-regulated | IGT vs ND |
| AC012370.3 | 2.0719 | 0.0064 | up-regulated | IGT vs ND |
| ANXA2R | 2.0518 | 0.0301 | up-regulated | IGT vs ND |
| RP11-171G2.1 | 2.0481 | 0.0410 | up-regulated | IGT vs ND |
| UGT2B4 | 2.0407 | 0.0389 | up-regulated | IGT vs ND |
| TTLL10-AS1 | 2.0388 | 0.0063 | up-regulated | IGT vs ND |
| CTD-2306A12.1 | 2.0224 | 0.0446 | up-regulated | IGT vs ND |
| LRRD1 | 2.0195 | 0.0134 | up-regulated | IGT vs ND |
| RARA-AS1 | 2.0132 | 0.0047 | up-regulated | IGT vs ND |
| KB-1507C5.3 | 1.9975 | 0.0451 | up-regulated | IGT vs ND |
| LINC01585 | 1.9960 | 0.0356 | up-regulated | IGT vs ND |
| NOX5 | 1.9879 | 0.0118 | up-regulated | IGT vs ND |
| SLC16A1-AS1 | 1.9879 | 0.0061 | up-regulated | IGT vs ND |
| ZFPM2-AS1 | 1.9841 | 0.0434 | up-regulated | IGT vs ND |
| RP11-359K18.4 | 1.9795 | 0.0431 | up-regulated | IGT vs ND |
| RP3-395M20.2 | 1.9769 | 0.0117 | up-regulated | IGT vs ND |
| ZNF625 | 1.9614 | 0.0340 | up-regulated | IGT vs ND |
| RP11-278A23.1 | 1.9553 | 0.0478 | up-regulated | IGT vs ND |
| ZIM2-AS1 | 1.9382 | 0.0171 | up-regulated | IGT vs ND |
| AC003956.1 | 1.9345 | 0.0271 | up-regulated | IGT vs ND |
| AC096670.3 | 1.9299 | 0.0019 | up-regulated | IGT vs ND |
| LDHAL6A | 1.9299 | 0.0010 | up-regulated | IGT vs ND |
| AP4B1-AS1 | 1.9293 | 0.0055 | up-regulated | IGT vs ND |
| PRSS48 | 1.9236 | 0.0406 | up-regulated | IGT vs ND |
| RDH12 | 1.9210 | 0.0003 | up-regulated | IGT vs ND |
| CCDC38 | 1.9193 | 0.0138 | up-regulated | IGT vs ND |
| WNT5B | 1.9175 | 0.0131 | up-regulated | IGT vs ND |
| GPR132 | 1.9148 | 0.0333 | up-regulated | IGT vs ND |
| CTA-331P3.1 | 1.9124 | 0.0451 | up-regulated | IGT vs ND |
| FAM156A | 1.9089 | 0.0195 | up-regulated | IGT vs ND |
| CTC-332L22.1 | 1.9080 | 0.0192 | up-regulated | IGT vs ND |
| AC005009.2 | 1.9069 | 0.0140 | up-regulated | IGT vs ND |
| CTC-492K19.7 | 1.9000 | 0.0058 | up-regulated | IGT vs ND |
| C6orf58 | 1.8931 | 0.0014 | up-regulated | IGT vs ND |
| AC004543.2 | 1.8924 | 0.0326 | up-regulated | IGT vs ND |
| RP11-115D19.3 | 1.8907 | 0.0372 | up-regulated | IGT vs ND |
| BIN2 | 1.8844 | 0.0254 | up-regulated | IGT vs ND |
| SLFN12 | 1.8813 | 0.0118 | up-regulated | IGT vs ND |
| C9orf173-AS1 | 1.8803 | 0.0240 | up-regulated | IGT vs ND |
| RP11-45M22.3 | 1.8755 | 0.0280 | up-regulated | IGT vs ND |
| ACKR2 | 1.8747 | 0.0346 | up-regulated | IGT vs ND |
| RELN | 1.8724 | 0.0294 | up-regulated | IGT vs ND |
| CXorf21 | 1.8694 | 0.0494 | up-regulated | IGT vs ND |
| LINC00106 | 1.8646 | 0.0078 | up-regulated | IGT vs ND |
| KB-1000E4.2 | 1.8537 | 0.0458 | up-regulated | IGT vs ND |
| PI16 | 1.8528 | 0.0381 | up-regulated | IGT vs ND |
| PZP | 1.8484 | 0.0174 | up-regulated | IGT vs ND |
| CASS4 | 1.8396 | 0.0060 | up-regulated | IGT vs ND |
| RP11-264B17.2 | 1.8392 | 0.0103 | up-regulated | IGT vs ND |
| CDH13 | 1.8239 | 0.0429 | up-regulated | IGT vs ND |
| LA16c-429E7.1 | 1.8168 | 0.0143 | up-regulated | IGT vs ND |
| NAT8 | 1.8148 | 0.0009 | up-regulated | IGT vs ND |
| DPEP2 | 1.8111 | 0.0345 | up-regulated | IGT vs ND |
| RP11-718O11.1 | 1.8063 | 0.0260 | up-regulated | IGT vs ND |
| SUCNR1 | 1.8057 | 0.0481 | up-regulated | IGT vs ND |
| GRIK3 | 1.8021 | 0.0334 | up-regulated | IGT vs ND |
| ACTA2 | 1.7993 | 0.0128 | up-regulated | IGT vs ND |
| NDNF | 1.7931 | 0.0323 | up-regulated | IGT vs ND |
| RP11-214K3.22 | 1.7877 | 0.0119 | up-regulated | IGT vs ND |
| C9orf106 | 1.7825 | 0.0224 | up-regulated | IGT vs ND |
| PLP1 | 1.7729 | 0.0146 | up-regulated | IGT vs ND |
| RP11-154J22.1 | 1.7712 | 0.0009 | up-regulated | IGT vs ND |
| HLA-DQA2 | 1.7645 | 0.0471 | up-regulated | IGT vs ND |
| RP11-301N24.3 | 1.7571 | 0.0320 | up-regulated | IGT vs ND |
| AC000403.4 | 1.7536 | 0.0279 | up-regulated | IGT vs ND |
| CYSLTR2 | 1.7348 | 0.0400 | up-regulated | IGT vs ND |
| C15orf56 | 1.7340 | 0.0089 | up-regulated | IGT vs ND |
| RP11-47J17.2 | 1.7181 | 0.0419 | up-regulated | IGT vs ND |
| RP1-269M15.3 | 1.6997 | 0.0139 | up-regulated | IGT vs ND |
| RP11-676J12.4 | 1.6969 | 0.0424 | up-regulated | IGT vs ND |
| RP11-458J1.1 | 1.6968 | 0.0318 | up-regulated | IGT vs ND |
| ASGR1 | 1.6944 | 0.0005 | up-regulated | IGT vs ND |
| RP4-633O19__A.1 | 1.6942 | 0.0331 | up-regulated | IGT vs ND |
| MYCBPAP | 1.6834 | 0.0089 | up-regulated | IGT vs ND |
| SP140 | 1.6813 | 0.0217 | up-regulated | IGT vs ND |
| CCNB2 | 1.6702 | 0.0334 | up-regulated | IGT vs ND |
| PITRM1-AS1 | 1.6689 | 0.0061 | up-regulated | IGT vs ND |
| C10orf95 | 1.6680 | 0.0233 | up-regulated | IGT vs ND |
| SFRP4 | 1.6666 | 0.0334 | up-regulated | IGT vs ND |
| KB-1460A1.1 | 1.6624 | 0.0498 | up-regulated | IGT vs ND |
| RAI1-AS1 | 1.6593 | 0.0436 | up-regulated | IGT vs ND |
| C2 | 1.6470 | 0.0021 | up-regulated | IGT vs ND |
| RELT | 1.6400 | 0.0371 | up-regulated | IGT vs ND |
| C1orf186 | 1.6336 | 0.0031 | up-regulated | IGT vs ND |
| SYTL5 | 1.6274 | 0.0239 | up-regulated | IGT vs ND |
| SHISA2 | 1.6261 | 0.0097 | up-regulated | IGT vs ND |
| ARHGEF6 | 1.6254 | 0.0070 | up-regulated | IGT vs ND |
| RP11-192H23.4 | 1.6155 | 0.0121 | up-regulated | IGT vs ND |
| GNA15 | 1.6146 | 0.0468 | up-regulated | IGT vs ND |
| OPRM1 | 1.6045 | 0.0284 | up-regulated | IGT vs ND |
| LMOD1 | 1.5962 | 0.0265 | up-regulated | IGT vs ND |
| RASL11A | 1.5956 | 0.0212 | up-regulated | IGT vs ND |
| RP11-368I7.6 | 1.5907 | 0.0112 | up-regulated | IGT vs ND |
| CD1D | 1.5892 | 0.0226 | up-regulated | IGT vs ND |
| VIP | 1.5875 | 0.0108 | up-regulated | IGT vs ND |
| KLHL6 | 1.5868 | 0.0116 | up-regulated | IGT vs ND |
| RIN3 | 1.5777 | 0.0006 | up-regulated | IGT vs ND |
| FGB | 1.5713 | 0.0420 | up-regulated | IGT vs ND |
| PARP10 | 1.5571 | 0.0020 | up-regulated | IGT vs ND |
| AC005740.5 | 1.5352 | 0.0371 | up-regulated | IGT vs ND |
| DOCK2 | 1.5325 | 0.0308 | up-regulated | IGT vs ND |
| MIR181A1HG | 1.5277 | 0.0287 | up-regulated | IGT vs ND |
| APOL3 | 1.5248 | 0.0056 | up-regulated | IGT vs ND |
| RP11-165F24.3 | 1.5213 | 0.0459 | up-regulated | IGT vs ND |
| RP11-1148O4.2 | 1.5200 | 0.0145 | up-regulated | IGT vs ND |
| ADAMTS4 | 1.5122 | 0.0085 | up-regulated | IGT vs ND |
| GBP5 | 1.5118 | 0.0268 | up-regulated | IGT vs ND |
| TRAIP | 1.5046 | 0.0032 | up-regulated | IGT vs ND |
| PDZRN4 | 1.5029 | 0.0044 | up-regulated | IGT vs ND |
| RP5-1074L1.1 | 1.5004 | 0.0297 | up-regulated | IGT vs ND |
| RP11-996F15.4 | 1.4881 | 0.0018 | up-regulated | IGT vs ND |
| TMEM191A | 1.4846 | 0.0492 | up-regulated | IGT vs ND |
| VWA5B1 | 1.4816 | 0.0432 | up-regulated | IGT vs ND |
| HCP5B | 1.4787 | 0.0132 | up-regulated | IGT vs ND |
| SMC1B | 1.4779 | 0.0187 | up-regulated | IGT vs ND |
| RP11-449G16.1 | 1.4690 | 0.0098 | up-regulated | IGT vs ND |
| RP11-638I8.1 | 1.4658 | 0.0019 | up-regulated | IGT vs ND |
| GPR124 | 1.4534 | 0.0053 | up-regulated | IGT vs ND |
| RP11-129I19.2 | 1.4513 | 0.0205 | up-regulated | IGT vs ND |
| RNASEH2B-AS1 | 1.4500 | 0.0066 | up-regulated | IGT vs ND |
| DCC | 1.4434 | 0.0153 | up-regulated | IGT vs ND |
| COL4A5 | 1.4413 | 0.0311 | up-regulated | IGT vs ND |
| BHLHE40-AS1 | 1.4405 | 0.0213 | up-regulated | IGT vs ND |
| CRISPLD1 | 1.4335 | 0.0296 | up-regulated | IGT vs ND |
| TRAF3IP3 | 1.4276 | 0.0200 | up-regulated | IGT vs ND |
| TRPV2 | 1.4181 | 0.0133 | up-regulated | IGT vs ND |
| SERTM1 | 1.4168 | 0.0019 | up-regulated | IGT vs ND |
| TPSAB1 | 1.4151 | 0.0146 | up-regulated | IGT vs ND |
| PTGES2-AS1 | 1.4150 | 0.0013 | up-regulated | IGT vs ND |
| HTR2B | 1.4124 | 0.0047 | up-regulated | IGT vs ND |
| SHISA7 | 1.4091 | 0.0078 | up-regulated | IGT vs ND |
| IL18R1 | 1.4063 | 0.0310 | up-regulated | IGT vs ND |
| RP11-210K20.2 | 1.4027 | 0.0448 | up-regulated | IGT vs ND |
| SLMO1 | 1.4017 | 0.0271 | up-regulated | IGT vs ND |
| TMPRSS11D | 1.4013 | 0.0146 | up-regulated | IGT vs ND |
| RP11-728G15.1 | 1.4006 | 0.0364 | up-regulated | IGT vs ND |
| RP11-754B17.1 | 1.3987 | 0.0325 | up-regulated | IGT vs ND |
| CYTH4 | 1.3973 | 0.0145 | up-regulated | IGT vs ND |
| RP11-88H10.2 | 1.3927 | 0.0064 | up-regulated | IGT vs ND |
| RP11-15A1.2 | 1.3881 | 0.0000 | up-regulated | IGT vs ND |
| FAM81A | 1.3706 | 0.0276 | up-regulated | IGT vs ND |
| PRTG | 1.3686 | 0.0283 | up-regulated | IGT vs ND |
| PDGFRB | 1.3680 | 0.0302 | up-regulated | IGT vs ND |
| RP11-500G22.5 | 1.3573 | 0.0280 | up-regulated | IGT vs ND |
| IL16 | 1.3527 | 0.0239 | up-regulated | IGT vs ND |
| BRINP1 | 1.3507 | 0.0402 | up-regulated | IGT vs ND |
| PTPRE | 1.3474 | 0.0010 | up-regulated | IGT vs ND |
| TMPRSS12 | 1.3468 | 0.0294 | up-regulated | IGT vs ND |
| CTA-223H9.9 | 1.3457 | 0.0462 | up-regulated | IGT vs ND |
| TAGLN | 1.3414 | 0.0272 | up-regulated | IGT vs ND |
| CTD-3064M3.7 | 1.3397 | 0.0440 | up-regulated | IGT vs ND |
| ZNF90 | 1.3392 | 0.0337 | up-regulated | IGT vs ND |
| GPR112 | 1.3305 | 0.0475 | up-regulated | IGT vs ND |
| AOC3 | 1.3288 | 0.0224 | up-regulated | IGT vs ND |
| THSD1 | 1.3191 | 0.0050 | up-regulated | IGT vs ND |
| RP11-44N11.1 | 1.3172 | 0.0411 | up-regulated | IGT vs ND |
| ARHGAP6 | 1.3150 | 0.0396 | up-regulated | IGT vs ND |
| NXPH3 | 1.3112 | 0.0171 | up-regulated | IGT vs ND |
| CRLF2 | 1.3100 | 0.0136 | up-regulated | IGT vs ND |
| AC018816.3 | 1.3063 | 0.0157 | up-regulated | IGT vs ND |
| RP11-527L4.2 | 1.3043 | 0.0001 | up-regulated | IGT vs ND |
| GGT5 | 1.3031 | 0.0414 | up-regulated | IGT vs ND |
| RASL10B | 1.2916 | 0.0265 | up-regulated | IGT vs ND |
| TPTEP1 | 1.2885 | 0.0313 | up-regulated | IGT vs ND |
| RP11-368I7.4 | 1.2841 | 0.0376 | up-regulated | IGT vs ND |
| STAB2 | 1.2764 | 0.0242 | up-regulated | IGT vs ND |
| PCDHGB7 | 1.2564 | 0.0315 | up-regulated | IGT vs ND |
| C14orf39 | 1.2531 | 0.0003 | up-regulated | IGT vs ND |
| ZNF346-IT1 | 1.2467 | 0.0180 | up-regulated | IGT vs ND |
| EVA1B | 1.2421 | 0.0204 | up-regulated | IGT vs ND |
| FRMPD3 | 1.2351 | 0.0010 | up-regulated | IGT vs ND |
| ST3GAL5-AS1 | 1.2301 | 0.0426 | up-regulated | IGT vs ND |
| POLR2J4 | 1.2289 | 0.0038 | up-regulated | IGT vs ND |
| MAZ | 1.2212 | 0.0472 | up-regulated | IGT vs ND |
| KCNA2 | 1.2164 | 0.0151 | up-regulated | IGT vs ND |
| OTUB2 | 1.2142 | 0.0270 | up-regulated | IGT vs ND |
| FERMT1 | 1.2122 | 0.0453 | up-regulated | IGT vs ND |
| RP11-244N9.6 | 1.2112 | 0.0141 | up-regulated | IGT vs ND |
| SPAG5 | 1.2080 | 0.0002 | up-regulated | IGT vs ND |
| C2CD4A | 1.2079 | 0.0342 | up-regulated | IGT vs ND |
| NGFR | 1.2034 | 0.0296 | up-regulated | IGT vs ND |
| AC083884.8 | 1.2022 | 0.0079 | up-regulated | IGT vs ND |
| RP11-443B7.1 | 1.2012 | 0.0462 | up-regulated | IGT vs ND |
| NUDT17 | 1.1942 | 0.0031 | up-regulated | IGT vs ND |
| KB-431C1.5 | 1.1904 | 0.0422 | up-regulated | IGT vs ND |
| TRPV3 | 1.1880 | 0.0238 | up-regulated | IGT vs ND |
| RP11-548P2.2 | 1.1854 | 0.0166 | up-regulated | IGT vs ND |
| AL133245.2 | 1.1824 | 0.0232 | up-regulated | IGT vs ND |
| GLYCTK | 1.1804 | 0.0000 | up-regulated | IGT vs ND |
| RP11-845C23.2 | 1.1712 | 0.0058 | up-regulated | IGT vs ND |
| MFGE8 | 1.1678 | 0.0198 | up-regulated | IGT vs ND |
| C1orf220 | 1.1676 | 0.0088 | up-regulated | IGT vs ND |
| BSN-AS2 | 1.1586 | 0.0262 | up-regulated | IGT vs ND |
| CYSRT1 | 1.1467 | 0.0170 | up-regulated | IGT vs ND |
| SMCR5 | 1.1417 | 0.0131 | up-regulated | IGT vs ND |
| KNTC1 | 1.1405 | 0.0007 | up-regulated | IGT vs ND |
| SLC14A1 | 1.1387 | 0.0191 | up-regulated | IGT vs ND |
| CPNE5 | 1.1387 | 0.0325 | up-regulated | IGT vs ND |
| AC002511.2 | 1.1334 | 0.0020 | up-regulated | IGT vs ND |
| MCAM | 1.1331 | 0.0491 | up-regulated | IGT vs ND |
| RAB34 | 1.1270 | 0.0394 | up-regulated | IGT vs ND |
| CBLC | 1.1209 | 0.0041 | up-regulated | IGT vs ND |
| SPDYE1 | 1.1121 | 0.0436 | up-regulated | IGT vs ND |
| AC084809.2 | 1.1096 | 0.0158 | up-regulated | IGT vs ND |
| PIGR | 1.1089 | 0.0436 | up-regulated | IGT vs ND |
| LILRB3 | 1.1061 | 0.0006 | up-regulated | IGT vs ND |
| PDCD4-AS1 | 1.1056 | 0.0037 | up-regulated | IGT vs ND |
| VSTM2A | 1.1022 | 0.0282 | up-regulated | IGT vs ND |
| PDE1C | 1.1019 | 0.0297 | up-regulated | IGT vs ND |
| RGS16 | 1.0884 | 0.0269 | up-regulated | IGT vs ND |
| PCDHGA9 | 1.0835 | 0.0244 | up-regulated | IGT vs ND |
| PKN3 | 1.0821 | 0.0445 | up-regulated | IGT vs ND |
| MLPH | 1.0618 | 0.0315 | up-regulated | IGT vs ND |
| AC098820.3 | 1.0616 | 0.0020 | up-regulated | IGT vs ND |
| MMP9 | 1.0540 | 0.0213 | up-regulated | IGT vs ND |
| LCP1 | 1.0532 | 0.0375 | up-regulated | IGT vs ND |
| ARHGEF10 | 1.0525 | 0.0348 | up-regulated | IGT vs ND |
| MYH11 | 1.0517 | 0.0255 | up-regulated | IGT vs ND |
| RP11-380B4.3 | 1.0491 | 0.0258 | up-regulated | IGT vs ND |
| RP4-639F20.1 | 1.0485 | 0.0033 | up-regulated | IGT vs ND |
| RP11-347C18.5 | 1.0459 | 0.0373 | up-regulated | IGT vs ND |
| HSD17B13 | 1.0432 | 0.0351 | up-regulated | IGT vs ND |
| ASIC1 | 1.0428 | 0.0233 | up-regulated | IGT vs ND |
| IL3RA | 1.0406 | 0.0012 | up-regulated | IGT vs ND |
| RASGRP3 | 1.0333 | 0.0366 | up-regulated | IGT vs ND |
| MYL9 | 1.0233 | 0.0319 | up-regulated | IGT vs ND |
| RP5-1021I20.1 | 1.0220 | 0.0398 | up-regulated | IGT vs ND |
| ACOT4 | 1.0174 | 0.0423 | up-regulated | IGT vs ND |
| RP11-799M12.2 | 1.0066 | 0.0383 | up-regulated | IGT vs ND |
| RBP2 | 1.0039 | 0.0369 | up-regulated | IGT vs ND |
| CTD-2005H7.2 | 1.0035 | 0.0492 | up-regulated | IGT vs ND |
| ELK3 | 1.0017 | 0.0433 | up-regulated | IGT vs ND |
| RP11-272P10.2 | -1.0282 | 0.0162 | down-regulated | IGT vs ND |
| RP11-70L8.4 | -1.0314 | 0.0454 | down-regulated | IGT vs ND |
| PHGDH | -1.0579 | 0.0213 | down-regulated | IGT vs ND |
| UGT2B7 | -1.0643 | 0.0433 | down-regulated | IGT vs ND |
| TSPAN19 | -1.0662 | 0.0128 | down-regulated | IGT vs ND |
| PCDHB3 | -1.0761 | 0.0045 | down-regulated | IGT vs ND |
| AC002550.6 | -1.0794 | 0.0311 | down-regulated | IGT vs ND |
| NEK10 | -1.0828 | 0.0003 | down-regulated | IGT vs ND |
| AGR2 | -1.0873 | 0.0471 | down-regulated | IGT vs ND |
| HSPA5 | -1.1012 | 0.0000 | down-regulated | IGT vs ND |
| GIPC2 | -1.1054 | 0.0490 | down-regulated | IGT vs ND |
| LINC01389 | -1.1110 | 0.0290 | down-regulated | IGT vs ND |
| RP11-44F21.5 | -1.1181 | 0.0177 | down-regulated | IGT vs ND |
| RP1-20B11.2 | -1.1332 | 0.0293 | down-regulated | IGT vs ND |
| UGT2A3 | -1.1426 | 0.0157 | down-regulated | IGT vs ND |
| SLC4A4 | -1.1572 | 0.0441 | down-regulated | IGT vs ND |
| NUP210L | -1.2124 | 0.0411 | down-regulated | IGT vs ND |
| FAM19A4 | -1.2270 | 0.0203 | down-regulated | IGT vs ND |
| FUT9 | -1.2457 | 0.0083 | down-regulated | IGT vs ND |
| RP1-122O8.7 | -1.2582 | 0.0340 | down-regulated | IGT vs ND |
| RP11-211N8.2 | -1.2585 | 0.0197 | down-regulated | IGT vs ND |
| RP11-503N18.1 | -1.3025 | 0.0462 | down-regulated | IGT vs ND |
| RP11-341N2.1 | -1.3071 | 0.0232 | down-regulated | IGT vs ND |
| GATM | -1.3227 | 0.0439 | down-regulated | IGT vs ND |
| FLJ38122 | -1.3341 | 0.0438 | down-regulated | IGT vs ND |
| CBX2 | -1.3362 | 0.0300 | down-regulated | IGT vs ND |
| RP4-613B23.1 | -1.3972 | 0.0192 | down-regulated | IGT vs ND |
| RP11-729I10.2 | -1.4089 | 0.0440 | down-regulated | IGT vs ND |
| CLDN10 | -1.4457 | 0.0361 | down-regulated | IGT vs ND |
| TNNI1 | -1.4492 | 0.0063 | down-regulated | IGT vs ND |
| FGFR2 | -1.4570 | 0.0221 | down-regulated | IGT vs ND |
| SLC19A1 | -1.4760 | 0.0128 | down-regulated | IGT vs ND |
| RP11-307O13.1 | -1.4801 | 0.0381 | down-regulated | IGT vs ND |
| TSIX | -1.4915 | 0.0360 | down-regulated | IGT vs ND |
| CUZD1 | -1.5076 | 0.0360 | down-regulated | IGT vs ND |
| CTD-2325P2.4 | -1.5283 | 0.0209 | down-regulated | IGT vs ND |
| PSAT1 | -1.5428 | 0.0340 | down-regulated | IGT vs ND |
| RP11-596D21.1 | -1.5676 | 0.0350 | down-regulated | IGT vs ND |
| TGFA | -1.5849 | 0.0355 | down-regulated | IGT vs ND |
| NEFL | -1.5874 | 0.0428 | down-regulated | IGT vs ND |
| RP11-110I1.14 | -1.5938 | 0.0177 | down-regulated | IGT vs ND |
| RP11-517B11.4 | -1.5985 | 0.0297 | down-regulated | IGT vs ND |
| ALB | -1.6063 | 0.0275 | down-regulated | IGT vs ND |
| A4GNT | -1.6125 | 0.0487 | down-regulated | IGT vs ND |
| RP11-427L15.2 | -1.6241 | 0.0410 | down-regulated | IGT vs ND |
| EGF | -1.6808 | 0.0221 | down-regulated | IGT vs ND |
| MYZAP | -1.6966 | 0.0228 | down-regulated | IGT vs ND |
| CFTR | -1.6991 | 0.0092 | down-regulated | IGT vs ND |
| BCAT1 | -1.6992 | 0.0160 | down-regulated | IGT vs ND |
| PSG4 | -1.7002 | 0.0176 | down-regulated | IGT vs ND |
| RP1-296L11.1 | -1.7301 | 0.0127 | down-regulated | IGT vs ND |
| TFPI2 | -1.7352 | 0.0232 | down-regulated | IGT vs ND |
| SPINK2 | -1.7491 | 0.0001 | down-regulated | IGT vs ND |
| TACSTD2 | -1.7547 | 0.0393 | down-regulated | IGT vs ND |
| RP11-415F23.3 | -1.7927 | 0.0239 | down-regulated | IGT vs ND |
| SERPINI2 | -1.8312 | 0.0192 | down-regulated | IGT vs ND |
| CCDC73 | -1.8499 | 0.0142 | down-regulated | IGT vs ND |
| RP11-181B11.2 | -1.8634 | 0.0211 | down-regulated | IGT vs ND |
| EPN3 | -1.8991 | 0.0308 | down-regulated | IGT vs ND |
| CTD-2215E18.3 | -2.0071 | 0.0478 | down-regulated | IGT vs ND |
| GUCA1C | -2.0303 | 0.0320 | down-regulated | IGT vs ND |
| DNLZ | -2.0368 | 0.0157 | down-regulated | IGT vs ND |
| RP11-428J1.4 | -2.0977 | 0.0077 | down-regulated | IGT vs ND |
| AADAC | -2.0989 | 0.0078 | down-regulated | IGT vs ND |
| GDNF | -2.1063 | 0.0391 | down-regulated | IGT vs ND |
| FGL1 | -2.1436 | 0.0094 | down-regulated | IGT vs ND |
| AC004549.6 | -2.1887 | 0.0021 | down-regulated | IGT vs ND |
| CACNA1F | -2.2193 | 0.0051 | down-regulated | IGT vs ND |
| FLT3 | -2.2965 | 0.0425 | down-regulated | IGT vs ND |
| LINC00222 | -2.3019 | 0.0193 | down-regulated | IGT vs ND |
| IGSF11-AS1 | -2.3126 | 0.0140 | down-regulated | IGT vs ND |
| LPAR3 | -2.3902 | 0.0116 | down-regulated | IGT vs ND |
| RP11-47P18.1 | -2.4584 | 0.0143 | down-regulated | IGT vs ND |
| AGR3 | -2.5512 | 0.0034 | down-regulated | IGT vs ND |
| UPK1B | -2.5799 | 0.0134 | down-regulated | IGT vs ND |
| KCNJ10 | 2.4354 | 0.0180 | up-regulated | DEP vs IGT |
| C15orf54 | 2.3816 | 0.0248 | up-regulated | DEP vs IGT |
| FLJ33360 | 2.2945 | 0.0010 | up-regulated | DEP vs IGT |
| CD180 | 2.2798 | 0.0026 | up-regulated | DEP vs IGT |
| SNX31 | 2.1734 | 0.0054 | up-regulated | DEP vs IGT |
| RP11-47P18.1 | 2.1293 | 0.0169 | up-regulated | DEP vs IGT |
| PNLDC1 | 2.1181 | 0.0314 | up-regulated | DEP vs IGT |
| OLFM4 | 2.0968 | 0.0036 | up-regulated | DEP vs IGT |
| PCDHA7 | 2.0435 | 0.0019 | up-regulated | DEP vs IGT |
| RP11-473M20.16 | 2.0342 | 0.0123 | up-regulated | DEP vs IGT |
| FGL1 | 2.0124 | 0.0139 | up-regulated | DEP vs IGT |
| B2M | 2.0111 | 0.0197 | up-regulated | DEP vs IGT |
| SLC35G5 | 2.0074 | 0.0234 | up-regulated | DEP vs IGT |
| GDPD4 | 1.9765 | 0.0374 | up-regulated | DEP vs IGT |
| KRT80 | 1.9668 | 0.0001 | up-regulated | DEP vs IGT |
| CFAP58 | 1.9128 | 0.0077 | up-regulated | DEP vs IGT |
| ART3 | 1.9120 | 0.0026 | up-regulated | DEP vs IGT |
| TJP2 | 1.9086 | 0.0079 | up-regulated | DEP vs IGT |
| AC093901.1 | 1.9067 | 0.0398 | up-regulated | DEP vs IGT |
| SERPINI2 | 1.9002 | 0.0114 | up-regulated | DEP vs IGT |
| PSAT1 | 1.8789 | 0.0010 | up-regulated | DEP vs IGT |
| WDR72 | 1.8704 | 0.0021 | up-regulated | DEP vs IGT |
| EGF | 1.8437 | 0.0170 | up-regulated | DEP vs IGT |
| AGR2 | 1.8408 | 0.0002 | up-regulated | DEP vs IGT |
| FOXD2-AS1 | 1.8385 | 0.0207 | up-regulated | DEP vs IGT |
| RP11-90L1.8 | 1.8152 | 0.0139 | up-regulated | DEP vs IGT |
| REG3A | 1.8032 | 0.0273 | up-regulated | DEP vs IGT |
| CNTF | 1.7668 | 0.0313 | up-regulated | DEP vs IGT |
| CXCL14 | 1.7532 | 0.0318 | up-regulated | DEP vs IGT |
| CTRB2 | 1.7522 | 0.0182 | up-regulated | DEP vs IGT |
| TACSTD2 | 1.7477 | 0.0181 | up-regulated | DEP vs IGT |
| RP11-44N22.3 | 1.7455 | 0.0282 | up-regulated | DEP vs IGT |
| SNCA-AS1 | 1.7209 | 0.0406 | up-regulated | DEP vs IGT |
| MIR3140 | 1.7103 | 0.0321 | up-regulated | DEP vs IGT |
| RP11-404P21.3 | 1.6982 | 0.0312 | up-regulated | DEP vs IGT |
| RP11-65J21.3 | 1.6969 | 0.0176 | up-regulated | DEP vs IGT |
| CUZD1 | 1.6815 | 0.0120 | up-regulated | DEP vs IGT |
| FAM217A | 1.6703 | 0.0118 | up-regulated | DEP vs IGT |
| ANKRD62 | 1.6588 | 0.0106 | up-regulated | DEP vs IGT |
| AADAC | 1.6584 | 0.0182 | up-regulated | DEP vs IGT |
| RP11-415F23.3 | 1.6374 | 0.0417 | up-regulated | DEP vs IGT |
| SYCN | 1.6364 | 0.0289 | up-regulated | DEP vs IGT |
| AQP8 | 1.6323 | 0.0227 | up-regulated | DEP vs IGT |
| PQLC2L | 1.6131 | 0.0049 | up-regulated | DEP vs IGT |
| PROM1 | 1.6065 | 0.0240 | up-regulated | DEP vs IGT |
| CCDC144A | 1.5967 | 0.0047 | up-regulated | DEP vs IGT |
| RP11-214K3.24 | 1.5940 | 0.0006 | up-regulated | DEP vs IGT |
| RP11-82L18.2 | 1.5876 | 0.0270 | up-regulated | DEP vs IGT |
| VNN2 | 1.5528 | 0.0187 | up-regulated | DEP vs IGT |
| RP11-305O6.4 | 1.5503 | 0.0089 | up-regulated | DEP vs IGT |
| FOXS1 | 1.5369 | 0.0214 | up-regulated | DEP vs IGT |
| RP11-320N7.2 | 1.5355 | 0.0372 | up-regulated | DEP vs IGT |
| AC006116.15 | 1.5308 | 0.0071 | up-regulated | DEP vs IGT |
| GLIS3-AS1 | 1.5294 | 0.0001 | up-regulated | DEP vs IGT |
| CYP26B1 | 1.5195 | 0.0257 | up-regulated | DEP vs IGT |
| CRNDE | 1.5078 | 0.0209 | up-regulated | DEP vs IGT |
| FREM2 | 1.5047 | 0.0169 | up-regulated | DEP vs IGT |
| SERHL | 1.4851 | 0.0089 | up-regulated | DEP vs IGT |
| FGFRL1 | 1.4831 | 0.0086 | up-regulated | DEP vs IGT |
| FLG2 | 1.4811 | 0.0060 | up-regulated | DEP vs IGT |
| PGM5P3-AS1 | 1.4793 | 0.0227 | up-regulated | DEP vs IGT |
| REG3G | 1.4793 | 0.0097 | up-regulated | DEP vs IGT |
| ARMC3 | 1.4774 | 0.0324 | up-regulated | DEP vs IGT |
| FGFR2 | 1.4769 | 0.0110 | up-regulated | DEP vs IGT |
| BCAT1 | 1.4760 | 0.0143 | up-regulated | DEP vs IGT |
| RP11-588H23.3 | 1.4736 | 0.0483 | up-regulated | DEP vs IGT |
| TYRO3 | 1.4718 | 0.0096 | up-regulated | DEP vs IGT |
| CFTR | 1.4712 | 0.0065 | up-regulated | DEP vs IGT |
| PLEKHS1 | 1.4688 | 0.0133 | up-regulated | DEP vs IGT |
| SEMA3B | 1.4645 | 0.0298 | up-regulated | DEP vs IGT |
| CCDC64B | 1.4530 | 0.0170 | up-regulated | DEP vs IGT |
| SLC4A4 | 1.4397 | 0.0036 | up-regulated | DEP vs IGT |
| SYCP2 | 1.4349 | 0.0002 | up-regulated | DEP vs IGT |
| RP11-557J10.4 | 1.4326 | 0.0201 | up-regulated | DEP vs IGT |
| VWA8-AS1 | 1.4255 | 0.0215 | up-regulated | DEP vs IGT |
| ISM1 | 1.4164 | 0.0361 | up-regulated | DEP vs IGT |
| KCNJ5 | 1.4143 | 0.0266 | up-regulated | DEP vs IGT |
| VTCN1 | 1.4124 | 0.0453 | up-regulated | DEP vs IGT |
| ACKR2 | 1.4102 | 0.0309 | up-regulated | DEP vs IGT |
| RP11-65L3.4 | 1.4073 | 0.0358 | up-regulated | DEP vs IGT |
| SERINC2 | 1.4039 | 0.0039 | up-regulated | DEP vs IGT |
| RP4-545K15.5 | 1.4033 | 0.0019 | up-regulated | DEP vs IGT |
| RP11-680B3.2 | 1.4027 | 0.0438 | up-regulated | DEP vs IGT |
| RP11-490G2.2 | 1.3984 | 0.0350 | up-regulated | DEP vs IGT |
| RP11-172H24.4 | 1.3935 | 0.0196 | up-regulated | DEP vs IGT |
| RP11-181E10.3 | 1.3802 | 0.0334 | up-regulated | DEP vs IGT |
| TSIX | 1.3623 | 0.0377 | up-regulated | DEP vs IGT |
| MYZAP | 1.3591 | 0.0354 | up-regulated | DEP vs IGT |
| FLJ38122 | 1.3522 | 0.0485 | up-regulated | DEP vs IGT |
| CTD-2325P2.4 | 1.3436 | 0.0490 | up-regulated | DEP vs IGT |
| FBXW12 | 1.3408 | 0.0143 | up-regulated | DEP vs IGT |
| ZMYND10 | 1.3219 | 0.0295 | up-regulated | DEP vs IGT |
| SDC1 | 1.3181 | 0.0332 | up-regulated | DEP vs IGT |
| RP11-70L8.4 | 1.3149 | 0.0125 | up-regulated | DEP vs IGT |
| AC008063.2 | 1.3106 | 0.0029 | up-regulated | DEP vs IGT |
| KANK4 | 1.3045 | 0.0375 | up-regulated | DEP vs IGT |
| ACADL | 1.2823 | 0.0220 | up-regulated | DEP vs IGT |
| PLIN5 | 1.2787 | 0.0297 | up-regulated | DEP vs IGT |
| CLDN10 | 1.2751 | 0.0322 | up-regulated | DEP vs IGT |
| PRSS16 | 1.2748 | 0.0060 | up-regulated | DEP vs IGT |
| RP11-408N14.1 | 1.2714 | 0.0224 | up-regulated | DEP vs IGT |
| TGFB2 | 1.2556 | 0.0253 | up-regulated | DEP vs IGT |
| PHGDH | 1.2493 | 0.0064 | up-regulated | DEP vs IGT |
| PCDHA11 | 1.2482 | 0.0311 | up-regulated | DEP vs IGT |
| TMEM217 | 1.2457 | 0.0440 | up-regulated | DEP vs IGT |
| PDZK1IP1 | 1.2435 | 0.0488 | up-regulated | DEP vs IGT |
| TMEM97 | 1.2267 | 0.0044 | up-regulated | DEP vs IGT |
| SLC17A4 | 1.2260 | 0.0050 | up-regulated | DEP vs IGT |
| TPST2 | 1.2216 | 0.0251 | up-regulated | DEP vs IGT |
| RP11-748C4.1 | 1.2177 | 0.0157 | up-regulated | DEP vs IGT |
| GATM | 1.2138 | 0.0457 | up-regulated | DEP vs IGT |
| MUC1 | 1.2077 | 0.0348 | up-regulated | DEP vs IGT |
| TREH | 1.2042 | 0.0460 | up-regulated | DEP vs IGT |
| RP11-392B6.1 | 1.2005 | 0.0153 | up-regulated | DEP vs IGT |
| P2RY8 | 1.1956 | 0.0379 | up-regulated | DEP vs IGT |
| FLRT2 | 1.1942 | 0.0020 | up-regulated | DEP vs IGT |
| GRHL2 | 1.1864 | 0.0211 | up-regulated | DEP vs IGT |
| SMAD9-IT1 | 1.1602 | 0.0398 | up-regulated | DEP vs IGT |
| LINC00671 | 1.1482 | 0.0461 | up-regulated | DEP vs IGT |
| RP11-359J14.2 | 1.1453 | 0.0119 | up-regulated | DEP vs IGT |
| SPAG17 | 1.1363 | 0.0328 | up-regulated | DEP vs IGT |
| PTENP1-AS | 1.1344 | 0.0091 | up-regulated | DEP vs IGT |
| RP11-39M21.2 | 1.1287 | 0.0492 | up-regulated | DEP vs IGT |
| CTD-2227E11.1 | 1.1278 | 0.0209 | up-regulated | DEP vs IGT |
| IL32 | 1.1243 | 0.0220 | up-regulated | DEP vs IGT |
| RP11-504A18.1 | 1.1193 | 0.0448 | up-regulated | DEP vs IGT |
| FGF12-AS2 | 1.1157 | 0.0478 | up-regulated | DEP vs IGT |
| OSBPL5 | 1.1102 | 0.0150 | up-regulated | DEP vs IGT |
| RP11-45P15.4 | 1.1068 | 0.0169 | up-regulated | DEP vs IGT |
| RP11-483C6.1 | 1.1067 | 0.0303 | up-regulated | DEP vs IGT |
| TAS2R30 | 1.1054 | 0.0312 | up-regulated | DEP vs IGT |
| SFRP5 | 1.1052 | 0.0368 | up-regulated | DEP vs IGT |
| FIRRE | 1.1023 | 0.0369 | up-regulated | DEP vs IGT |
| NIPAL1 | 1.0772 | 0.0030 | up-regulated | DEP vs IGT |
| RGS10 | 1.0678 | 0.0252 | up-regulated | DEP vs IGT |
| B3GNT7 | 1.0564 | 0.0042 | up-regulated | DEP vs IGT |
| ACOT4 | 1.0520 | 0.0017 | up-regulated | DEP vs IGT |
| SLC43A1 | 1.0428 | 0.0089 | up-regulated | DEP vs IGT |
| GDF15 | 1.0423 | 0.0133 | up-regulated | DEP vs IGT |
| FAM129A | 1.0416 | 0.0275 | up-regulated | DEP vs IGT |
| CA2 | 1.0395 | 0.0139 | up-regulated | DEP vs IGT |
| RP11-723O4.9 | 1.0385 | 0.0230 | up-regulated | DEP vs IGT |
| ARHGAP28 | 1.0346 | 0.0171 | up-regulated | DEP vs IGT |
| MANF | 1.0266 | 0.0000 | up-regulated | DEP vs IGT |
| PDGFD | 1.0220 | 0.0213 | up-regulated | DEP vs IGT |
| RP11-499F3.2 | 1.0021 | 0.0423 | up-regulated | DEP vs IGT |
| RP11-354P11.4 | -1.0068 | 0.0449 | down-regulated | DEP vs IGT |
| SERTM1 | -1.0072 | 0.0197 | down-regulated | DEP vs IGT |
| LINC01521 | -1.0228 | 0.0372 | down-regulated | DEP vs IGT |
| TSSK3 | -1.0340 | 0.0373 | down-regulated | DEP vs IGT |
| RP11-313P18.1 | -1.0352 | 0.0226 | down-regulated | DEP vs IGT |
| CPA5 | -1.0443 | 0.0020 | down-regulated | DEP vs IGT |
| RP11-579D7.4 | -1.0557 | 0.0166 | down-regulated | DEP vs IGT |
| RP11-318A15.8 | -1.0598 | 0.0101 | down-regulated | DEP vs IGT |
| HAUS7 | -1.0616 | 0.0276 | down-regulated | DEP vs IGT |
| NLRP2 | -1.0637 | 0.0265 | down-regulated | DEP vs IGT |
| ALG1L2 | -1.0762 | 0.0494 | down-regulated | DEP vs IGT |
| RP1-117O3.2 | -1.0796 | 0.0352 | down-regulated | DEP vs IGT |
| FRMD6-AS1 | -1.0837 | 0.0274 | down-regulated | DEP vs IGT |
| UNQ6494 | -1.0924 | 0.0426 | down-regulated | DEP vs IGT |
| RP11-46A10.5 | -1.0947 | 0.0106 | down-regulated | DEP vs IGT |
| STIL | -1.1030 | 0.0189 | down-regulated | DEP vs IGT |
| LRAT | -1.1036 | 0.0131 | down-regulated | DEP vs IGT |
| GCNT7 | -1.1123 | 0.0400 | down-regulated | DEP vs IGT |
| RP11-582J16.5 | -1.1213 | 0.0094 | down-regulated | DEP vs IGT |
| RP11-54O7.3 | -1.1237 | 0.0401 | down-regulated | DEP vs IGT |
| FAM212B-AS1 | -1.1293 | 0.0313 | down-regulated | DEP vs IGT |
| ARHGAP39 | -1.1300 | 0.0192 | down-regulated | DEP vs IGT |
| GRPEL2-AS1 | -1.1369 | 0.0257 | down-regulated | DEP vs IGT |
| RP4-673M15.1 | -1.1401 | 0.0497 | down-regulated | DEP vs IGT |
| RP11-367G18.1 | -1.1535 | 0.0425 | down-regulated | DEP vs IGT |
| CTD-2649C14.2 | -1.1554 | 0.0244 | down-regulated | DEP vs IGT |
| GDF9 | -1.1567 | 0.0057 | down-regulated | DEP vs IGT |
| SATL1 | -1.1661 | 0.0165 | down-regulated | DEP vs IGT |
| CTD-2574D22.2 | -1.1733 | 0.0198 | down-regulated | DEP vs IGT |
| MYOCD | -1.1844 | 0.0378 | down-regulated | DEP vs IGT |
| SH2D6 | -1.1848 | 0.0164 | down-regulated | DEP vs IGT |
| RP11-435O5.7 | -1.1908 | 0.0196 | down-regulated | DEP vs IGT |
| CDIPT-AS1 | -1.1916 | 0.0116 | down-regulated | DEP vs IGT |
| RP11-80H5.9 | -1.1953 | 0.0005 | down-regulated | DEP vs IGT |
| RP5-933K21.3 | -1.1991 | 0.0479 | down-regulated | DEP vs IGT |
| ADAMTS8 | -1.2201 | 0.0423 | down-regulated | DEP vs IGT |
| FAM85B | -1.2263 | 0.0255 | down-regulated | DEP vs IGT |
| RP11-437J2.3 | -1.2296 | 0.0330 | down-regulated | DEP vs IGT |
| AC013733.3 | -1.2320 | 0.0002 | down-regulated | DEP vs IGT |
| CEBPB-AS1 | -1.2335 | 0.0145 | down-regulated | DEP vs IGT |
| KCNH1-IT1 | -1.2364 | 0.0179 | down-regulated | DEP vs IGT |
| FAM81A | -1.2478 | 0.0105 | down-regulated | DEP vs IGT |
| RP11-120K24.5 | -1.2758 | 0.0331 | down-regulated | DEP vs IGT |
| CTD-2026K11.5 | -1.2788 | 0.0434 | down-regulated | DEP vs IGT |
| CTD-2313J17.5 | -1.2845 | 0.0457 | down-regulated | DEP vs IGT |
| RP11-304L19.11 | -1.2849 | 0.0029 | down-regulated | DEP vs IGT |
| AC137932.5 | -1.3188 | 0.0387 | down-regulated | DEP vs IGT |
| ALK | -1.3191 | 0.0189 | down-regulated | DEP vs IGT |
| RP5-963E22.6 | -1.3274 | 0.0338 | down-regulated | DEP vs IGT |
| RP11-293M10.6 | -1.3279 | 0.0072 | down-regulated | DEP vs IGT |
| CTD-3138B18.6 | -1.3475 | 0.0087 | down-regulated | DEP vs IGT |
| KCND3-AS1 | -1.3520 | 0.0057 | down-regulated | DEP vs IGT |
| OCA2 | -1.3522 | 0.0147 | down-regulated | DEP vs IGT |
| MYCN | -1.3559 | 0.0371 | down-regulated | DEP vs IGT |
| MIR300 | -1.3574 | 0.0001 | down-regulated | DEP vs IGT |
| CACNA1I | -1.3605 | 0.0382 | down-regulated | DEP vs IGT |
| FRMD5 | -1.3608 | 0.0116 | down-regulated | DEP vs IGT |
| RP11-326A19.3 | -1.3687 | 0.0460 | down-regulated | DEP vs IGT |
| AC005355.1 | -1.3710 | 0.0184 | down-regulated | DEP vs IGT |
| RP11-950C14.7 | -1.3911 | 0.0374 | down-regulated | DEP vs IGT |
| XRCC3 | -1.3958 | 0.0180 | down-regulated | DEP vs IGT |
| RAC3 | -1.4174 | 0.0212 | down-regulated | DEP vs IGT |
| SLC19A3 | -1.4189 | 0.0062 | down-regulated | DEP vs IGT |
| BATF3 | -1.4243 | 0.0495 | down-regulated | DEP vs IGT |
| UNC5A | -1.4331 | 0.0135 | down-regulated | DEP vs IGT |
| PCDH8 | -1.4373 | 0.0456 | down-regulated | DEP vs IGT |
| STRC | -1.4384 | 0.0241 | down-regulated | DEP vs IGT |
| SLC9C2 | -1.4599 | 0.0229 | down-regulated | DEP vs IGT |
| RP11-191G24.1 | -1.4731 | 0.0401 | down-regulated | DEP vs IGT |
| MYO18B | -1.4732 | 0.0147 | down-regulated | DEP vs IGT |
| RP11-21L23.3 | -1.4759 | 0.0416 | down-regulated | DEP vs IGT |
| S100A12 | -1.4858 | 0.0318 | down-regulated | DEP vs IGT |
| FOXM1 | -1.4863 | 0.0377 | down-regulated | DEP vs IGT |
| EPHA5 | -1.4865 | 0.0252 | down-regulated | DEP vs IGT |
| RP4-535B20.1 | -1.5077 | 0.0128 | down-regulated | DEP vs IGT |
| RP11-162A12.2 | -1.5099 | 0.0043 | down-regulated | DEP vs IGT |
| SNX32 | -1.5110 | 0.0443 | down-regulated | DEP vs IGT |
| GABRA2 | -1.5220 | 0.0096 | down-regulated | DEP vs IGT |
| SLC16A13 | -1.5239 | 0.0204 | down-regulated | DEP vs IGT |
| CDH20 | -1.5457 | 0.0014 | down-regulated | DEP vs IGT |
| ZNF625 | -1.5490 | 0.0353 | down-regulated | DEP vs IGT |
| RP11-728G15.1 | -1.5567 | 0.0070 | down-regulated | DEP vs IGT |
| U73166.2 | -1.5654 | 0.0160 | down-regulated | DEP vs IGT |
| RP11-363J20.1 | -1.5695 | 0.0036 | down-regulated | DEP vs IGT |
| CH507-154B10.2 | -1.5789 | 0.0155 | down-regulated | DEP vs IGT |
| MIR578 | -1.5884 | 0.0289 | down-regulated | DEP vs IGT |
| CXorf58 | -1.5965 | 0.0274 | down-regulated | DEP vs IGT |
| CACNA1F | -1.5967 | 0.0363 | down-regulated | DEP vs IGT |
| CARTPT | -1.6011 | 0.0132 | down-regulated | DEP vs IGT |
| RP11-666A8.7 | -1.6037 | 0.0416 | down-regulated | DEP vs IGT |
| AC009473.1 | -1.6113 | 0.0496 | down-regulated | DEP vs IGT |
| AC004543.2 | -1.6299 | 0.0292 | down-regulated | DEP vs IGT |
| RP1-168L15.5 | -1.6361 | 0.0198 | down-regulated | DEP vs IGT |
| CTD-2089N3.3 | -1.6436 | 0.0173 | down-regulated | DEP vs IGT |
| RP11-287F9.2 | -1.6538 | 0.0460 | down-regulated | DEP vs IGT |
| NCBP2L | -1.6550 | 0.0042 | down-regulated | DEP vs IGT |
| UGT2B4 | -1.6589 | 0.0430 | down-regulated | DEP vs IGT |
| CALB2 | -1.6721 | 0.0272 | down-regulated | DEP vs IGT |
| CTD-2587H19.3 | -1.6898 | 0.0054 | down-regulated | DEP vs IGT |
| LINC00540 | -1.7273 | 0.0471 | down-regulated | DEP vs IGT |
| RP13-516M14.2 | -1.7312 | 0.0122 | down-regulated | DEP vs IGT |
| SOX11 | -1.7391 | 0.0082 | down-regulated | DEP vs IGT |
| GPR112 | -1.7481 | 0.0068 | down-regulated | DEP vs IGT |
| AP4B1-AS1 | -1.7864 | 0.0022 | down-regulated | DEP vs IGT |
| KB-1460A1.3 | -1.7880 | 0.0490 | down-regulated | DEP vs IGT |
| AC007365.3 | -1.7925 | 0.0201 | down-regulated | DEP vs IGT |
| MIR4263 | -1.7937 | 0.0161 | down-regulated | DEP vs IGT |
| RP11-982M15.8 | -1.8039 | 0.0473 | down-regulated | DEP vs IGT |
| LINC00942 | -1.8159 | 0.0464 | down-regulated | DEP vs IGT |
| RP11-171G2.1 | -1.8242 | 0.0157 | down-regulated | DEP vs IGT |
| GPR85 | -1.8251 | 0.0350 | down-regulated | DEP vs IGT |
| RP11-429A20.4 | -1.8595 | 0.0293 | down-regulated | DEP vs IGT |
| CTD-2537I9.12 | -1.8596 | 0.0407 | down-regulated | DEP vs IGT |
| TAAR1 | -1.8600 | 0.0179 | down-regulated | DEP vs IGT |
| PILRB | -1.8722 | 0.0008 | down-regulated | DEP vs IGT |
| SGCA | -1.8758 | 0.0294 | down-regulated | DEP vs IGT |
| CTC-492K19.7 | -1.8759 | 0.0006 | down-regulated | DEP vs IGT |
| RP11-478P10.1 | -1.8932 | 0.0443 | down-regulated | DEP vs IGT |
| LTK | -1.8980 | 0.0377 | down-regulated | DEP vs IGT |
| FAM228A | -1.9030 | 0.0402 | down-regulated | DEP vs IGT |
| CAMKV | -1.9540 | 0.0196 | down-regulated | DEP vs IGT |
| RP11-215P8.4 | -1.9839 | 0.0485 | down-regulated | DEP vs IGT |
| ISX | -2.0030 | 0.0156 | down-regulated | DEP vs IGT |
| GPRC5D | -2.0118 | 0.0014 | down-regulated | DEP vs IGT |
| RP11-278A23.1 | -2.0219 | 0.0302 | down-regulated | DEP vs IGT |
| MOCOS | -2.0353 | 0.0052 | down-regulated | DEP vs IGT |
| CTA-331P3.1 | -2.0432 | 0.0184 | down-regulated | DEP vs IGT |
| TREM1 | -2.0510 | 0.0204 | down-regulated | DEP vs IGT |
| EGFLAM | -2.0544 | 0.0002 | down-regulated | DEP vs IGT |
| AC005523.3 | -2.0622 | 0.0236 | down-regulated | DEP vs IGT |
| GHRL | -2.0811 | 0.0031 | down-regulated | DEP vs IGT |
| RP11-993B23.3 | -2.1461 | 0.0049 | down-regulated | DEP vs IGT |
| CDC20 | -2.1511 | 0.0162 | down-regulated | DEP vs IGT |
| LINC00482 | -2.1649 | 0.0044 | down-regulated | DEP vs IGT |
| LDLRAD4-AS1 | -2.1919 | 0.0004 | down-regulated | DEP vs IGT |
| RASA2-IT1 | -2.1937 | 0.0193 | down-regulated | DEP vs IGT |
| RP11-476H24.1 | -2.2298 | 0.0078 | down-regulated | DEP vs IGT |
| RP11-379C10.4 | -2.2435 | 0.0149 | down-regulated | DEP vs IGT |
| KB-1000E4.2 | -2.2460 | 0.0079 | down-regulated | DEP vs IGT |
| RP11-158M2.3 | -2.2713 | 0.0097 | down-regulated | DEP vs IGT |
| RP11-295D4.1 | -2.2753 | 0.0148 | down-regulated | DEP vs IGT |
| LILRA2 | -2.2953 | 0.0058 | down-regulated | DEP vs IGT |
| RP11-701P16.2 | -2.2966 | 0.0080 | down-regulated | DEP vs IGT |
| RENBP | -2.3052 | 0.0198 | down-regulated | DEP vs IGT |
| IL4 | -2.3852 | 0.0016 | down-regulated | DEP vs IGT |
| C8orf86 | -2.4372 | 0.0160 | down-regulated | DEP vs IGT |
| RP11-156L14.1 | -2.7233 | 0.0030 | down-regulated | DEP vs IGT |
| IL4I1 | -2.7549 | 0.0023 | down-regulated | DEP vs IGT |
| TSPEAR-AS1 | -2.7722 | 0.0052 | down-regulated | DEP vs IGT |
| CRISP3 | -2.8007 | 0.0008 | down-regulated | DEP vs IGT |
| CNTN2 | -2.8095 | 0.0033 | down-regulated | DEP vs IGT |
| RP11-479O17.10 | -2.8142 | 0.0000 | down-regulated | DEP vs IGT |
| RP11-679B17.2 | -3.2684 | 0.0010 | down-regulated | DEP vs IGT |
| RP11-298J23.5 | -3.3400 | 0.0010 | down-regulated | DEP vs IGT |
| TPX2 | 4.0103 | 0.0056 | up-regulated | DEP vs ND |
| PLIN4 | 3.8418 | 0.0000 | up-regulated | DEP vs ND |
| IL12RB2 | 3.7830 | 0.0003 | up-regulated | DEP vs ND |
| TAC4 | 3.5636 | 0.0320 | up-regulated | DEP vs ND |
| PDCD1LG2 | 3.5438 | 0.0048 | up-regulated | DEP vs ND |
| LAX1 | 3.4959 | 0.0017 | up-regulated | DEP vs ND |
| NCCRP1 | 3.4832 | 0.0104 | up-regulated | DEP vs ND |
| RP11-336A10.5 | 3.4510 | 0.0056 | up-regulated | DEP vs ND |
| GIMAP5 | 3.4066 | 0.0000 | up-regulated | DEP vs ND |
| RP11-1069G10.1 | 3.3280 | 0.0003 | up-regulated | DEP vs ND |
| RP11-63G10.3 | 3.3114 | 0.0161 | up-regulated | DEP vs ND |
| ACKR2 | 3.2818 | 0.0004 | up-regulated | DEP vs ND |
| COL17A1 | 3.2701 | 0.0083 | up-regulated | DEP vs ND |
| SIGLEC1 | 3.2078 | 0.0057 | up-regulated | DEP vs ND |
| NFE2 | 3.2058 | 0.0196 | up-regulated | DEP vs ND |
| C17orf53 | 3.1939 | 0.0024 | up-regulated | DEP vs ND |
| MYH15 | 3.1929 | 0.0200 | up-regulated | DEP vs ND |
| CXCL13 | 3.1454 | 0.0053 | up-regulated | DEP vs ND |
| SAA1 | 3.1240 | 0.0073 | up-regulated | DEP vs ND |
| ADIPOQ | 3.0682 | 0.0079 | up-regulated | DEP vs ND |
| SLC22A7 | 2.9876 | 0.0122 | up-regulated | DEP vs ND |
| RP4-536B24.3 | 2.9836 | 0.0258 | up-regulated | DEP vs ND |
| CTD-2306A12.1 | 2.9689 | 0.0044 | up-regulated | DEP vs ND |
| OASL | 2.9633 | 0.0109 | up-regulated | DEP vs ND |
| RP11-185E12.2 | 2.9324 | 0.0239 | up-regulated | DEP vs ND |
| BIN2 | 2.8529 | 0.0000 | up-regulated | DEP vs ND |
| TTLL10-AS1 | 2.8527 | 0.0001 | up-regulated | DEP vs ND |
| TNFSF15 | 2.8424 | 0.0009 | up-regulated | DEP vs ND |
| CEACAM7 | 2.8363 | 0.0072 | up-regulated | DEP vs ND |
| IGSF23 | 2.8283 | 0.0435 | up-regulated | DEP vs ND |
| MEP1B | 2.8227 | 0.0020 | up-regulated | DEP vs ND |
| HSPB7 | 2.7917 | 0.0306 | up-regulated | DEP vs ND |
| AC009784.3 | 2.7855 | 0.0128 | up-regulated | DEP vs ND |
| SIGLEC8 | 2.7751 | 0.0205 | up-regulated | DEP vs ND |
| LINC01235 | 2.7655 | 0.0116 | up-regulated | DEP vs ND |
| B2M | 2.7594 | 0.0176 | up-regulated | DEP vs ND |
| C8orf31 | 2.7517 | 0.0128 | up-regulated | DEP vs ND |
| ITGB6 | 2.7487 | 0.0060 | up-regulated | DEP vs ND |
| GFI1B | 2.7463 | 0.0272 | up-regulated | DEP vs ND |
| NXPE4 | 2.7446 | 0.0393 | up-regulated | DEP vs ND |
| AC007349.7 | 2.7366 | 0.0128 | up-regulated | DEP vs ND |
| CD247 | 2.7131 | 0.0164 | up-regulated | DEP vs ND |
| RP11-1024P17.1 | 2.7114 | 0.0016 | up-regulated | DEP vs ND |
| AC007099.1 | 2.7090 | 0.0183 | up-regulated | DEP vs ND |
| CHRNB4 | 2.6958 | 0.0257 | up-regulated | DEP vs ND |
| RP11-139H15.5 | 2.6925 | 0.0121 | up-regulated | DEP vs ND |
| RP11-327O17.2 | 2.6922 | 0.0166 | up-regulated | DEP vs ND |
| RP11-399E6.1 | 2.6720 | 0.0170 | up-regulated | DEP vs ND |
| RP11-490G2.2 | 2.6544 | 0.0084 | up-regulated | DEP vs ND |
| THSD7B | 2.6462 | 0.0081 | up-regulated | DEP vs ND |
| CECR7 | 2.6385 | 0.0210 | up-regulated | DEP vs ND |
| HSF2BP | 2.6305 | 0.0324 | up-regulated | DEP vs ND |
| CTB-32O4.2 | 2.6233 | 0.0235 | up-regulated | DEP vs ND |
| MEIG1 | 2.6120 | 0.0036 | up-regulated | DEP vs ND |
| NOX5 | 2.5916 | 0.0093 | up-regulated | DEP vs ND |
| RP11-255H23.4 | 2.5908 | 0.0257 | up-regulated | DEP vs ND |
| STOX1 | 2.5658 | 0.0080 | up-regulated | DEP vs ND |
| RP11-651P23.5 | 2.5489 | 0.0077 | up-regulated | DEP vs ND |
| RP11-506K6.4 | 2.5433 | 0.0281 | up-regulated | DEP vs ND |
| TSPAN18 | 2.5421 | 0.0000 | up-regulated | DEP vs ND |
| CTC-332L22.1 | 2.5262 | 0.0198 | up-regulated | DEP vs ND |
| C21orf33 | 2.4738 | 0.0425 | up-regulated | DEP vs ND |
| RP11-524F11.1 | 2.4696 | 0.0446 | up-regulated | DEP vs ND |
| CTD-3193O13.12 | 2.4677 | 0.0254 | up-regulated | DEP vs ND |
| C3orf36 | 2.4592 | 0.0168 | up-regulated | DEP vs ND |
| P2RY8 | 2.4580 | 0.0003 | up-regulated | DEP vs ND |
| GPR132 | 2.4424 | 0.0093 | up-regulated | DEP vs ND |
| TMEM217 | 2.4380 | 0.0037 | up-regulated | DEP vs ND |
| BANF2 | 2.4350 | 0.0263 | up-regulated | DEP vs ND |
| HHIPL1 | 2.4344 | 0.0150 | up-regulated | DEP vs ND |
| RP11-403A21.1 | 2.4255 | 0.0320 | up-regulated | DEP vs ND |
| RP11-473M20.16 | 2.4202 | 0.0395 | up-regulated | DEP vs ND |
| CXorf21 | 2.4181 | 0.0325 | up-regulated | DEP vs ND |
| FAM217A | 2.4105 | 0.0188 | up-regulated | DEP vs ND |
| CD180 | 2.3935 | 0.0194 | up-regulated | DEP vs ND |
| SOCS1 | 2.3932 | 0.0106 | up-regulated | DEP vs ND |
| RP11-504A18.1 | 2.3650 | 0.0052 | up-regulated | DEP vs ND |
| GLDN | 2.3291 | 0.0058 | up-regulated | DEP vs ND |
| POSTN | 2.3220 | 0.0176 | up-regulated | DEP vs ND |
| AC106873.4 | 2.3120 | 0.0451 | up-regulated | DEP vs ND |
| RP3-395M20.2 | 2.3005 | 0.0041 | up-regulated | DEP vs ND |
| PGF | 2.2890 | 0.0037 | up-regulated | DEP vs ND |
| LDB3 | 2.2612 | 0.0066 | up-regulated | DEP vs ND |
| RP11-107D24.2 | 2.2530 | 0.0449 | up-regulated | DEP vs ND |
| TM4SF20 | 2.2443 | 0.0470 | up-regulated | DEP vs ND |
| SELPLG | 2.2438 | 0.0050 | up-regulated | DEP vs ND |
| RP11-181E10.3 | 2.2286 | 0.0074 | up-regulated | DEP vs ND |
| MKI67 | 2.2207 | 0.0319 | up-regulated | DEP vs ND |
| LDHAL6A | 2.2201 | 0.0048 | up-regulated | DEP vs ND |
| GRIA1 | 2.2169 | 0.0480 | up-regulated | DEP vs ND |
| BUB1 | 2.1966 | 0.0411 | up-regulated | DEP vs ND |
| CDH13 | 2.1964 | 0.0066 | up-regulated | DEP vs ND |
| RP11-557J10.4 | 2.1963 | 0.0240 | up-regulated | DEP vs ND |
| COL11A1 | 2.1851 | 0.0182 | up-regulated | DEP vs ND |
| NRARP | 2.1817 | 0.0381 | up-regulated | DEP vs ND |
| AP001627.1 | 2.1767 | 0.0402 | up-regulated | DEP vs ND |
| BMP7 | 2.1535 | 0.0173 | up-regulated | DEP vs ND |
| RP11-497E19.1 | 2.1477 | 0.0440 | up-regulated | DEP vs ND |
| RP11-473M20.9 | 2.1448 | 0.0244 | up-regulated | DEP vs ND |
| ELFN1 | 2.1329 | 0.0342 | up-regulated | DEP vs ND |
| SFRP4 | 2.1320 | 0.0032 | up-regulated | DEP vs ND |
| FAM156A | 2.1308 | 0.0499 | up-regulated | DEP vs ND |
| SLFN12 | 2.1195 | 0.0040 | up-regulated | DEP vs ND |
| RP4-568F9.6 | 2.1128 | 0.0265 | up-regulated | DEP vs ND |
| RP11-1148O4.2 | 2.1038 | 0.0033 | up-regulated | DEP vs ND |
| CPXM1 | 2.0943 | 0.0412 | up-regulated | DEP vs ND |
| ACOT4 | 2.0741 | 0.0000 | up-regulated | DEP vs ND |
| RP11-748C4.1 | 2.0729 | 0.0118 | up-regulated | DEP vs ND |
| LRIT3 | 2.0722 | 0.0187 | up-regulated | DEP vs ND |
| RP11-184D12.1 | 2.0664 | 0.0456 | up-regulated | DEP vs ND |
| RP11-301N24.3 | 2.0632 | 0.0331 | up-regulated | DEP vs ND |
| VWA8-AS1 | 2.0422 | 0.0123 | up-regulated | DEP vs ND |
| RASL10B | 2.0241 | 0.0052 | up-regulated | DEP vs ND |
| RP11-444E17.6 | 2.0088 | 0.0121 | up-regulated | DEP vs ND |
| RP11-386M24.3 | 2.0041 | 0.0434 | up-regulated | DEP vs ND |
| PBOV1 | 2.0013 | 0.0118 | up-regulated | DEP vs ND |
| RP11-585P4.5 | 1.9910 | 0.0457 | up-regulated | DEP vs ND |
| LLNLR-246C6.1 | 1.9898 | 0.0138 | up-regulated | DEP vs ND |
| RP11-47J17.2 | 1.9888 | 0.0330 | up-regulated | DEP vs ND |
| CTA-254O6.1 | 1.9827 | 0.0375 | up-regulated | DEP vs ND |
| APLNR | 1.9729 | 0.0070 | up-regulated | DEP vs ND |
| ADAMTS4 | 1.9699 | 0.0009 | up-regulated | DEP vs ND |
| TNK2-AS1 | 1.9694 | 0.0380 | up-regulated | DEP vs ND |
| RP4-591N18.2 | 1.9684 | 0.0432 | up-regulated | DEP vs ND |
| TPSAB1 | 1.9578 | 0.0025 | up-regulated | DEP vs ND |
| RP11-70D24.3 | 1.9376 | 0.0332 | up-regulated | DEP vs ND |
| GNA15 | 1.9375 | 0.0238 | up-regulated | DEP vs ND |
| C15orf53 | 1.9367 | 0.0453 | up-regulated | DEP vs ND |
| LEFTY1 | 1.9356 | 0.0156 | up-regulated | DEP vs ND |
| PITRM1-AS1 | 1.9307 | 0.0033 | up-regulated | DEP vs ND |
| RELN | 1.9186 | 0.0424 | up-regulated | DEP vs ND |
| RP11-354E11.2 | 1.9032 | 0.0126 | up-regulated | DEP vs ND |
| EGR3 | 1.9030 | 0.0138 | up-regulated | DEP vs ND |
| RP11-188P20.3 | 1.9018 | 0.0033 | up-regulated | DEP vs ND |
| DYSF | 1.9016 | 0.0236 | up-regulated | DEP vs ND |
| PKD1L1 | 1.8885 | 0.0498 | up-regulated | DEP vs ND |
| KLHL6 | 1.8830 | 0.0056 | up-regulated | DEP vs ND |
| WTIP | 1.8635 | 0.0115 | up-regulated | DEP vs ND |
| RP11-723O4.9 | 1.8574 | 0.0032 | up-regulated | DEP vs ND |
| RP11-540A21.2 | 1.8476 | 0.0224 | up-regulated | DEP vs ND |
| CTD-2626G11.2 | 1.8470 | 0.0233 | up-regulated | DEP vs ND |
| ADAM28 | 1.8385 | 0.0016 | up-regulated | DEP vs ND |
| RP11-392B6.1 | 1.8288 | 0.0073 | up-regulated | DEP vs ND |
| ORC1 | 1.8096 | 0.0464 | up-regulated | DEP vs ND |
| TMEM191A | 1.8056 | 0.0264 | up-regulated | DEP vs ND |
| RP11-214K3.22 | 1.8052 | 0.0413 | up-regulated | DEP vs ND |
| VWA5B1 | 1.7966 | 0.0384 | up-regulated | DEP vs ND |
| PGM5P3-AS1 | 1.7942 | 0.0381 | up-regulated | DEP vs ND |
| NRIP3 | 1.7875 | 0.0440 | up-regulated | DEP vs ND |
| RP5-1074L1.1 | 1.7826 | 0.0304 | up-regulated | DEP vs ND |
| RP11-736K20.5 | 1.7800 | 0.0348 | up-regulated | DEP vs ND |
| CASS4 | 1.7598 | 0.0306 | up-regulated | DEP vs ND |
| AC090616.2 | 1.7507 | 0.0414 | up-regulated | DEP vs ND |
| SORCS2 | 1.7484 | 0.0204 | up-regulated | DEP vs ND |
| HOXA-AS2 | 1.7446 | 0.0478 | up-regulated | DEP vs ND |
| PARP10 | 1.7357 | 0.0033 | up-regulated | DEP vs ND |
| ANGPTL4 | 1.7320 | 0.0447 | up-regulated | DEP vs ND |
| CST2 | 1.7229 | 0.0082 | up-regulated | DEP vs ND |
| HTR2B | 1.7211 | 0.0004 | up-regulated | DEP vs ND |
| NAT8 | 1.7209 | 0.0012 | up-regulated | DEP vs ND |
| DOCK2 | 1.7188 | 0.0083 | up-regulated | DEP vs ND |
| RP11-996F15.4 | 1.7174 | 0.0150 | up-regulated | DEP vs ND |
| CENPF | 1.7129 | 0.0090 | up-regulated | DEP vs ND |
| PCDHA7 | 1.7069 | 0.0391 | up-regulated | DEP vs ND |
| TRPV3 | 1.6959 | 0.0173 | up-regulated | DEP vs ND |
| TRPC6 | 1.6899 | 0.0060 | up-regulated | DEP vs ND |
| FCN3 | 1.6875 | 0.0064 | up-regulated | DEP vs ND |
| SLMO1 | 1.6782 | 0.0146 | up-regulated | DEP vs ND |
| VIP | 1.6730 | 0.0163 | up-regulated | DEP vs ND |
| CCRL2 | 1.6728 | 0.0387 | up-regulated | DEP vs ND |
| ITIH3 | 1.6708 | 0.0440 | up-regulated | DEP vs ND |
| SPAG5-AS1 | 1.6633 | 0.0190 | up-regulated | DEP vs ND |
| OXTR | 1.6603 | 0.0297 | up-regulated | DEP vs ND |
| ITGAM | 1.6398 | 0.0269 | up-regulated | DEP vs ND |
| NOTUM | 1.6393 | 0.0440 | up-regulated | DEP vs ND |
| LINC01465 | 1.6293 | 0.0157 | up-regulated | DEP vs ND |
| ITGB4 | 1.6291 | 0.0129 | up-regulated | DEP vs ND |
| GJC1 | 1.6190 | 0.0158 | up-regulated | DEP vs ND |
| SOSTDC1 | 1.6187 | 0.0167 | up-regulated | DEP vs ND |
| ARHGAP6 | 1.6147 | 0.0289 | up-regulated | DEP vs ND |
| IFI27 | 1.6110 | 0.0093 | up-regulated | DEP vs ND |
| HOTAIRM1 | 1.6089 | 0.0452 | up-regulated | DEP vs ND |
| PTAFR | 1.6047 | 0.0108 | up-regulated | DEP vs ND |
| PGM5P4-AS1 | 1.5988 | 0.0262 | up-regulated | DEP vs ND |
| FOSB | 1.5931 | 0.0431 | up-regulated | DEP vs ND |
| PDGFRB | 1.5913 | 0.0001 | up-regulated | DEP vs ND |
| C1orf186 | 1.5868 | 0.0022 | up-regulated | DEP vs ND |
| RP11-262H14.3 | 1.5834 | 0.0253 | up-regulated | DEP vs ND |
| CDH5 | 1.5820 | 0.0283 | up-regulated | DEP vs ND |
| ADAM33 | 1.5816 | 0.0328 | up-regulated | DEP vs ND |
| KCNA2 | 1.5732 | 0.0049 | up-regulated | DEP vs ND |
| PYGL | 1.5721 | 0.0063 | up-regulated | DEP vs ND |
| GBP4 | 1.5656 | 0.0085 | up-regulated | DEP vs ND |
| ARHGAP22 | 1.5641 | 0.0427 | up-regulated | DEP vs ND |
| RARG | 1.5600 | 0.0007 | up-regulated | DEP vs ND |
| GJA4 | 1.5558 | 0.0466 | up-regulated | DEP vs ND |
| DIO2 | 1.5551 | 0.0004 | up-regulated | DEP vs ND |
| FOXS1 | 1.5523 | 0.0494 | up-regulated | DEP vs ND |
| ITGAL | 1.5506 | 0.0432 | up-regulated | DEP vs ND |
| PRDM1 | 1.5505 | 0.0119 | up-regulated | DEP vs ND |
| RBP7 | 1.5487 | 0.0012 | up-regulated | DEP vs ND |
| IL34 | 1.5440 | 0.0385 | up-regulated | DEP vs ND |
| C2 | 1.5437 | 0.0134 | up-regulated | DEP vs ND |
| GPR124 | 1.5406 | 0.0080 | up-regulated | DEP vs ND |
| AC006129.2 | 1.5337 | 0.0387 | up-regulated | DEP vs ND |
| ARHGEF6 | 1.5306 | 0.0060 | up-regulated | DEP vs ND |
| RERGL | 1.5241 | 0.0185 | up-regulated | DEP vs ND |
| CMKLR1 | 1.5238 | 0.0092 | up-regulated | DEP vs ND |
| PIGR | 1.5192 | 0.0474 | up-regulated | DEP vs ND |
| GDF15 | 1.5123 | 0.0169 | up-regulated | DEP vs ND |
| RP4-647C14.3 | 1.5094 | 0.0095 | up-regulated | DEP vs ND |
| ALDOB | 1.5087 | 0.0051 | up-regulated | DEP vs ND |
| RDH12 | 1.4918 | 0.0007 | up-regulated | DEP vs ND |
| RASSF9 | 1.4909 | 0.0281 | up-regulated | DEP vs ND |
| SLFN13 | 1.4861 | 0.0365 | up-regulated | DEP vs ND |
| ADAMTS17 | 1.4737 | 0.0252 | up-regulated | DEP vs ND |
| NXPH3 | 1.4708 | 0.0418 | up-regulated | DEP vs ND |
| GLIS3-AS1 | 1.4659 | 0.0041 | up-regulated | DEP vs ND |
| CSRP2 | 1.4590 | 0.0112 | up-regulated | DEP vs ND |
| FOLR2 | 1.4583 | 0.0411 | up-regulated | DEP vs ND |
| TBX3 | 1.4567 | 0.0282 | up-regulated | DEP vs ND |
| HSD17B13 | 1.4521 | 0.0031 | up-regulated | DEP vs ND |
| TMPRSS11D | 1.4448 | 0.0220 | up-regulated | DEP vs ND |
| TRPV2 | 1.4425 | 0.0253 | up-regulated | DEP vs ND |
| COL8A1 | 1.4423 | 0.0255 | up-regulated | DEP vs ND |
| AC087294.2 | 1.4371 | 0.0322 | up-regulated | DEP vs ND |
| CLEC2B | 1.4356 | 0.0147 | up-regulated | DEP vs ND |
| CSPG4 | 1.4351 | 0.0337 | up-regulated | DEP vs ND |
| AC008063.2 | 1.4335 | 0.0401 | up-regulated | DEP vs ND |
| CPA3 | 1.4301 | 0.0320 | up-regulated | DEP vs ND |
| PKNOX2 | 1.4291 | 0.0335 | up-regulated | DEP vs ND |
| FGF1 | 1.4287 | 0.0237 | up-regulated | DEP vs ND |
| QRICH2 | 1.4282 | 0.0079 | up-regulated | DEP vs ND |
| RP5-1021I20.1 | 1.4281 | 0.0159 | up-regulated | DEP vs ND |
| RP5-1092A11.5 | 1.4260 | 0.0249 | up-regulated | DEP vs ND |
| PARVG | 1.4253 | 0.0033 | up-regulated | DEP vs ND |
| PRTG | 1.4162 | 0.0173 | up-regulated | DEP vs ND |
| RP11-190A12.7 | 1.4145 | 0.0110 | up-regulated | DEP vs ND |
| CTD-2033D15.2 | 1.4109 | 0.0435 | up-regulated | DEP vs ND |
| TNFRSF10D | 1.4100 | 0.0111 | up-regulated | DEP vs ND |
| SPAG5 | 1.4093 | 0.0074 | up-regulated | DEP vs ND |
| COL21A1 | 1.4045 | 0.0144 | up-regulated | DEP vs ND |
| PDE1C | 1.4022 | 0.0059 | up-regulated | DEP vs ND |
| RASGRP3 | 1.3955 | 0.0046 | up-regulated | DEP vs ND |
| EGR4 | 1.3847 | 0.0144 | up-regulated | DEP vs ND |
| GIMAP7 | 1.3721 | 0.0256 | up-regulated | DEP vs ND |
| TPSB2 | 1.3701 | 0.0489 | up-regulated | DEP vs ND |
| NDUFA4L2 | 1.3694 | 0.0329 | up-regulated | DEP vs ND |
| CRISPLD1 | 1.3660 | 0.0486 | up-regulated | DEP vs ND |
| ELK3 | 1.3640 | 0.0003 | up-regulated | DEP vs ND |
| CTSE | 1.3618 | 0.0451 | up-regulated | DEP vs ND |
| MFAP2 | 1.3600 | 0.0352 | up-regulated | DEP vs ND |
| RAMP3 | 1.3595 | 0.0003 | up-regulated | DEP vs ND |
| PODXL | 1.3515 | 0.0068 | up-regulated | DEP vs ND |
| C14orf39 | 1.3488 | 0.0004 | up-regulated | DEP vs ND |
| RP11-499F3.2 | 1.3486 | 0.0298 | up-regulated | DEP vs ND |
| SHISA7 | 1.3429 | 0.0323 | up-regulated | DEP vs ND |
| THSD1 | 1.3395 | 0.0374 | up-regulated | DEP vs ND |
| CD37 | 1.3375 | 0.0098 | up-regulated | DEP vs ND |
| CCDC96 | 1.3340 | 0.0051 | up-regulated | DEP vs ND |
| DBH-AS1 | 1.3333 | 0.0364 | up-regulated | DEP vs ND |
| BTNL9 | 1.3305 | 0.0071 | up-regulated | DEP vs ND |
| KDR | 1.3296 | 0.0354 | up-regulated | DEP vs ND |
| CASP10 | 1.3274 | 0.0019 | up-regulated | DEP vs ND |
| ESAM | 1.3264 | 0.0139 | up-regulated | DEP vs ND |
| SYTL4 | 1.3119 | 0.0341 | up-regulated | DEP vs ND |
| ENPEP | 1.3088 | 0.0212 | up-regulated | DEP vs ND |
| RP11-466A19.1 | 1.3064 | 0.0017 | up-regulated | DEP vs ND |
| NOTCH4 | 1.3057 | 0.0060 | up-regulated | DEP vs ND |
| PTPRE | 1.3055 | 0.0021 | up-regulated | DEP vs ND |
| COX4I2 | 1.3053 | 0.0111 | up-regulated | DEP vs ND |
| GIMAP6 | 1.2984 | 0.0177 | up-regulated | DEP vs ND |
| AURKC | 1.2976 | 0.0391 | up-regulated | DEP vs ND |
| TRAF3IP3 | 1.2928 | 0.0248 | up-regulated | DEP vs ND |
| ELTD1 | 1.2893 | 0.0289 | up-regulated | DEP vs ND |
| PLVAP | 1.2891 | 0.0318 | up-regulated | DEP vs ND |
| CXorf36 | 1.2857 | 0.0237 | up-regulated | DEP vs ND |
| RP11-380B4.3 | 1.2851 | 0.0386 | up-regulated | DEP vs ND |
| EPHB4 | 1.2830 | 0.0386 | up-regulated | DEP vs ND |
| SEPT4 | 1.2822 | 0.0038 | up-regulated | DEP vs ND |
| SYNDIG1L | 1.2808 | 0.0138 | up-regulated | DEP vs ND |
| BST2 | 1.2807 | 0.0046 | up-regulated | DEP vs ND |
| HIC1 | 1.2782 | 0.0218 | up-regulated | DEP vs ND |
| CPA4 | 1.2776 | 0.0127 | up-regulated | DEP vs ND |
| C8orf46 | 1.2763 | 0.0224 | up-regulated | DEP vs ND |
| RASSF2 | 1.2725 | 0.0185 | up-regulated | DEP vs ND |
| SCUBE2 | 1.2723 | 0.0298 | up-regulated | DEP vs ND |
| CHRDL1 | 1.2676 | 0.0446 | up-regulated | DEP vs ND |
| PLXDC1 | 1.2583 | 0.0283 | up-regulated | DEP vs ND |
| MMP9 | 1.2569 | 0.0328 | up-regulated | DEP vs ND |
| ACTA2 | 1.2567 | 0.0314 | up-regulated | DEP vs ND |
| RCOR2 | 1.2537 | 0.0164 | up-regulated | DEP vs ND |
| CD84 | 1.2525 | 0.0475 | up-regulated | DEP vs ND |
| IFITM1 | 1.2502 | 0.0312 | up-regulated | DEP vs ND |
| RP11-755F10.1 | 1.2492 | 0.0489 | up-regulated | DEP vs ND |
| GPR4 | 1.2447 | 0.0428 | up-regulated | DEP vs ND |
| MYL9 | 1.2428 | 0.0056 | up-regulated | DEP vs ND |
| EDNRA | 1.2392 | 0.0293 | up-regulated | DEP vs ND |
| CALCRL | 1.2387 | 0.0377 | up-regulated | DEP vs ND |
| CPNE5 | 1.2375 | 0.0328 | up-regulated | DEP vs ND |
| RP11-126K1.6 | 1.2358 | 0.0297 | up-regulated | DEP vs ND |
| RP4-545K15.5 | 1.2343 | 0.0427 | up-regulated | DEP vs ND |
| HMOX1 | 1.2338 | 0.0086 | up-regulated | DEP vs ND |
| RASD2 | 1.2308 | 0.0096 | up-regulated | DEP vs ND |
| CD248 | 1.2296 | 0.0207 | up-regulated | DEP vs ND |
| LAMA4 | 1.2279 | 0.0004 | up-regulated | DEP vs ND |
| VWF | 1.2260 | 0.0061 | up-regulated | DEP vs ND |
| LCP2 | 1.2252 | 0.0041 | up-regulated | DEP vs ND |
| ARHGEF10 | 1.2235 | 0.0281 | up-regulated | DEP vs ND |
| AC093495.4 | 1.2175 | 0.0171 | up-regulated | DEP vs ND |
| OLFML2A | 1.2109 | 0.0104 | up-regulated | DEP vs ND |
| STARD8 | 1.2090 | 0.0285 | up-regulated | DEP vs ND |
| ARHGDIB | 1.2041 | 0.0279 | up-regulated | DEP vs ND |
| BHLHE40 | 1.1999 | 0.0006 | up-regulated | DEP vs ND |
| RP3-395M20.8 | 1.1998 | 0.0430 | up-regulated | DEP vs ND |
| RP11-368I7.4 | 1.1943 | 0.0341 | up-regulated | DEP vs ND |
| KNTC1 | 1.1934 | 0.0029 | up-regulated | DEP vs ND |
| ASAP2 | 1.1923 | 0.0151 | up-regulated | DEP vs ND |
| FGF7 | 1.1911 | 0.0172 | up-regulated | DEP vs ND |
| AOC3 | 1.1909 | 0.0401 | up-regulated | DEP vs ND |
| ANO1 | 1.1902 | 0.0297 | up-regulated | DEP vs ND |
| CD34 | 1.1897 | 0.0391 | up-regulated | DEP vs ND |
| APOL3 | 1.1888 | 0.0351 | up-regulated | DEP vs ND |
| MLPH | 1.1856 | 0.0070 | up-regulated | DEP vs ND |
| IL3RA | 1.1854 | 0.0065 | up-regulated | DEP vs ND |
| HSPG2 | 1.1852 | 0.0054 | up-regulated | DEP vs ND |
| ITGA9 | 1.1834 | 0.0089 | up-regulated | DEP vs ND |
| GGT5 | 1.1804 | 0.0444 | up-regulated | DEP vs ND |
| LAPTM5 | 1.1673 | 0.0247 | up-regulated | DEP vs ND |
| EBF2 | 1.1590 | 0.0367 | up-regulated | DEP vs ND |
| SEMA6C | 1.1561 | 0.0307 | up-regulated | DEP vs ND |
| POLR2J4 | 1.1548 | 0.0190 | up-regulated | DEP vs ND |
| CRISPLD2 | 1.1469 | 0.0379 | up-regulated | DEP vs ND |
| FJX1 | 1.1464 | 0.0336 | up-regulated | DEP vs ND |
| APOC1 | 1.1455 | 0.0224 | up-regulated | DEP vs ND |
| MIR143HG | 1.1453 | 0.0452 | up-regulated | DEP vs ND |
| PDGFB | 1.1453 | 0.0042 | up-regulated | DEP vs ND |
| IFI44 | 1.1444 | 0.0271 | up-regulated | DEP vs ND |
| EBF1 | 1.1421 | 0.0108 | up-regulated | DEP vs ND |
| F12 | 1.1419 | 0.0065 | up-regulated | DEP vs ND |
| PRICKLE2-AS3 | 1.1371 | 0.0127 | up-regulated | DEP vs ND |
| PCDH12 | 1.1362 | 0.0003 | up-regulated | DEP vs ND |
| GNRHR | 1.1356 | 0.0213 | up-regulated | DEP vs ND |
| CTSS | 1.1295 | 0.0192 | up-regulated | DEP vs ND |
| IGSF10 | 1.1294 | 0.0436 | up-regulated | DEP vs ND |
| VSTM4 | 1.1276 | 0.0052 | up-regulated | DEP vs ND |
| UST | 1.1273 | 0.0372 | up-regulated | DEP vs ND |
| RAB34 | 1.1262 | 0.0246 | up-regulated | DEP vs ND |
| LGALS1 | 1.1175 | 0.0262 | up-regulated | DEP vs ND |
| SNAI3-AS1 | 1.1136 | 0.0102 | up-regulated | DEP vs ND |
| NIPAL1 | 1.1132 | 0.0037 | up-regulated | DEP vs ND |
| GIMAP4 | 1.1127 | 0.0120 | up-regulated | DEP vs ND |
| RGCC | 1.1099 | 0.0392 | up-regulated | DEP vs ND |
| IFIT3 | 1.1097 | 0.0007 | up-regulated | DEP vs ND |
| TMEM88 | 1.1059 | 0.0089 | up-regulated | DEP vs ND |
| HCP5B | 1.1044 | 0.0297 | up-regulated | DEP vs ND |
| INCENP | 1.1043 | 0.0494 | up-regulated | DEP vs ND |
| CDR2L | 1.1038 | 0.0317 | up-regulated | DEP vs ND |
| COL4A1 | 1.0991 | 0.0455 | up-regulated | DEP vs ND |
| DKK3 | 1.0948 | 0.0006 | up-regulated | DEP vs ND |
| ICAM1 | 1.0935 | 0.0499 | up-regulated | DEP vs ND |
| DARS-AS1 | 1.0932 | 0.0365 | up-regulated | DEP vs ND |
| KCNAB2 | 1.0888 | 0.0008 | up-regulated | DEP vs ND |
| RGS16 | 1.0880 | 0.0093 | up-regulated | DEP vs ND |
| ECSCR | 1.0870 | 0.0358 | up-regulated | DEP vs ND |
| MMRN2 | 1.0804 | 0.0394 | up-regulated | DEP vs ND |
| IMPDH1 | 1.0769 | 0.0107 | up-regulated | DEP vs ND |
| PTGES2-AS1 | 1.0758 | 0.0321 | up-regulated | DEP vs ND |
| SLCO2B1 | 1.0682 | 0.0343 | up-regulated | DEP vs ND |
| GLYCTK | 1.0666 | 0.0026 | up-regulated | DEP vs ND |
| TMEM47 | 1.0658 | 0.0045 | up-regulated | DEP vs ND |
| CD58 | 1.0649 | 0.0011 | up-regulated | DEP vs ND |
| TNFRSF12A | 1.0636 | 0.0068 | up-regulated | DEP vs ND |
| ARHGAP31 | 1.0594 | 0.0047 | up-regulated | DEP vs ND |
| MET | 1.0587 | 0.0466 | up-regulated | DEP vs ND |
| ROM1 | 1.0563 | 0.0061 | up-regulated | DEP vs ND |
| RP11-506H21.5 | 1.0561 | 0.0357 | up-regulated | DEP vs ND |
| PTPRC | 1.0534 | 0.0342 | up-regulated | DEP vs ND |
| STARD9 | 1.0515 | 0.0006 | up-regulated | DEP vs ND |
| FES | 1.0513 | 0.0446 | up-regulated | DEP vs ND |
| PTRF | 1.0510 | 0.0143 | up-regulated | DEP vs ND |
| ITGA5 | 1.0471 | 0.0035 | up-regulated | DEP vs ND |
| ENC1 | 1.0431 | 0.0123 | up-regulated | DEP vs ND |
| SLCO2A1 | 1.0424 | 0.0323 | up-regulated | DEP vs ND |
| PPAP2A | 1.0406 | 0.0033 | up-regulated | DEP vs ND |
| ZNF699 | 1.0367 | 0.0428 | up-regulated | DEP vs ND |
| UACA | 1.0366 | 0.0062 | up-regulated | DEP vs ND |
| TPH1 | 1.0323 | 0.0029 | up-regulated | DEP vs ND |
| RP11-699L21.1 | 1.0320 | 0.0260 | up-regulated | DEP vs ND |
| JAM2 | 1.0292 | 0.0122 | up-regulated | DEP vs ND |
| MXRA8 | 1.0278 | 0.0378 | up-regulated | DEP vs ND |
| COL6A2 | 1.0258 | 0.0213 | up-regulated | DEP vs ND |
| RHOC | 1.0092 | 0.0002 | up-regulated | DEP vs ND |
| SLC14A1 | 1.0085 | 0.0142 | up-regulated | DEP vs ND |
| GPX8 | 1.0028 | 0.0178 | up-regulated | DEP vs ND |
| ITPKB | 1.0017 | 0.0427 | up-regulated | DEP vs ND |
| UBXN2A | 1.0010 | 0.0000 | up-regulated | DEP vs ND |
| MMP28 | -1.0018 | 0.0262 | down-regulated | DEP vs ND |
| NUDT11 | -1.0199 | 0.0155 | down-regulated | DEP vs ND |
| RP11-166O4.6 | -1.0410 | 0.0259 | down-regulated | DEP vs ND |
| KANSL1-AS1 | -1.0627 | 0.0465 | down-regulated | DEP vs ND |
| RP11-92K15.1 | -1.0700 | 0.0292 | down-regulated | DEP vs ND |
| DLK1 | -1.1021 | 0.0018 | down-regulated | DEP vs ND |
| WDR24 | -1.1025 | 0.0115 | down-regulated | DEP vs ND |
| RP11-46A10.5 | -1.1136 | 0.0386 | down-regulated | DEP vs ND |
| IMPG2 | -1.1163 | 0.0365 | down-regulated | DEP vs ND |
| HRK | -1.1198 | 0.0486 | down-regulated | DEP vs ND |
| LRRC74A | -1.1927 | 0.0181 | down-regulated | DEP vs ND |
| RP11-1007O24.2 | -1.2040 | 0.0237 | down-regulated | DEP vs ND |
| NEK10 | -1.2155 | 0.0003 | down-regulated | DEP vs ND |
| AC010082.2 | -1.2821 | 0.0446 | down-regulated | DEP vs ND |
| RP11-367N14.3 | -1.2834 | 0.0125 | down-regulated | DEP vs ND |
| HIST3H2BB | -1.3052 | 0.0088 | down-regulated | DEP vs ND |
| RP11-154H23.3 | -1.3210 | 0.0460 | down-regulated | DEP vs ND |
| CTD-2012J19.3 | -1.3227 | 0.0240 | down-regulated | DEP vs ND |
| PACSIN1 | -1.3462 | 0.0162 | down-regulated | DEP vs ND |
| LRAT | -1.3467 | 0.0345 | down-regulated | DEP vs ND |
| MYOCD | -1.3708 | 0.0195 | down-regulated | DEP vs ND |
| RP11-385G16.1 | -1.3958 | 0.0420 | down-regulated | DEP vs ND |
| CARTPT | -1.4010 | 0.0464 | down-regulated | DEP vs ND |
| RP11-93G5.1 | -1.4380 | 0.0178 | down-regulated | DEP vs ND |
| PILRB | -1.4426 | 0.0103 | down-regulated | DEP vs ND |
| RP11-57C13.6 | -1.4502 | 0.0063 | down-regulated | DEP vs ND |
| FAM19A4 | -1.4516 | 0.0055 | down-regulated | DEP vs ND |
| TAS2R20 | -1.4708 | 0.0101 | down-regulated | DEP vs ND |
| SPINK2 | -1.5009 | 0.0042 | down-regulated | DEP vs ND |
| KIZ-AS1 | -1.5076 | 0.0072 | down-regulated | DEP vs ND |
| SULT1A2 | -1.5133 | 0.0214 | down-regulated | DEP vs ND |
| ORC6 | -1.5160 | 0.0158 | down-regulated | DEP vs ND |
| GRPEL2-AS1 | -1.5198 | 0.0465 | down-regulated | DEP vs ND |
| CTD-2007L18.5 | -1.5376 | 0.0331 | down-regulated | DEP vs ND |
| ACSM6 | -1.5444 | 0.0012 | down-regulated | DEP vs ND |
| RP1-117O3.2 | -1.5445 | 0.0069 | down-regulated | DEP vs ND |
| CTD-2026K11.5 | -1.5511 | 0.0394 | down-regulated | DEP vs ND |
| AC006116.24 | -1.6437 | 0.0333 | down-regulated | DEP vs ND |
| UNC5A | -1.6810 | 0.0165 | down-regulated | DEP vs ND |
| NUP210L | -1.7222 | 0.0099 | down-regulated | DEP vs ND |
| IL6 | -1.7727 | 0.0498 | down-regulated | DEP vs ND |
| RP11-145M4.3 | -1.8473 | 0.0474 | down-regulated | DEP vs ND |
| EGFLAM | -1.9214 | 0.0234 | down-regulated | DEP vs ND |
| RGPD2 | -1.9914 | 0.0114 | down-regulated | DEP vs ND |
| RP11-435J9.2 | -2.0466 | 0.0133 | down-regulated | DEP vs ND |
| TAAR1 | -2.0574 | 0.0443 | down-regulated | DEP vs ND |
| DNLZ | -2.0576 | 0.0290 | down-regulated | DEP vs ND |
| RP11-424N24.2 | -2.0583 | 0.0374 | down-regulated | DEP vs ND |
| IL4 | -2.0879 | 0.0181 | down-regulated | DEP vs ND |
| FCHO1 | -2.1133 | 0.0086 | down-regulated | DEP vs ND |
| CTD-2562G15.3 | -2.1493 | 0.0061 | down-regulated | DEP vs ND |
| RP11-429J17.7 | -2.1498 | 0.0287 | down-regulated | DEP vs ND |
| RP11-802D6.1 | -2.2358 | 0.0107 | down-regulated | DEP vs ND |
| AC006160.5 | -2.2372 | 0.0338 | down-regulated | DEP vs ND |
| CLPSL1 | -2.2809 | 0.0461 | down-regulated | DEP vs ND |
| AC104820.2 | -2.3082 | 0.0426 | down-regulated | DEP vs ND |
| RP11-517B11.4 | -2.3952 | 0.0104 | down-regulated | DEP vs ND |
| MIR635 | -2.4533 | 0.0328 | down-regulated | DEP vs ND |
| BRINP2 | -2.5534 | 0.0288 | down-regulated | DEP vs ND |
| PCDH8 | -2.5660 | 0.0021 | down-regulated | DEP vs ND |
| MOCOS | -2.6397 | 0.0025 | down-regulated | DEP vs ND |
| CTD-3064H18.4 | -2.7645 | 0.0152 | down-regulated | DEP vs ND |
| RP11-60A24.3 | -2.8214 | 0.0053 | down-regulated | DEP vs ND |
| LDLRAD4-AS1 | -2.8421 | 0.0023 | down-regulated | DEP vs ND |
| AC116609.3 | -2.9341 | 0.0101 | down-regulated | DEP vs ND |
| CAMKV | -2.9958 | 0.0006 | down-regulated | DEP vs ND |
| TSPEAR-AS1 | -3.2939 | 0.0107 | down-regulated | DEP vs ND |
| CDC20 | -3.6902 | 0.0051 | down-regulated | DEP vs ND |
| CACNA1F | -3.8259 | 0.0011 | down-regulated | DEP vs ND |
| RP11-298J23.5 | -3.9806 | 0.0008 | down-regulated | DEP vs ND |
