# Supplementary material for: Mechanism exploration and biomarker identification of glycemic deterioration in patients with diseases of the exocrine pancreas
Source: Sci Rep. 2024 Feb 22;14:4374. doi: 10.1038/s41598-024-52956-x (PMC10883946; doi:10.1038/s41598-024-52956-x)
Supplement: Supplementary file 11 — Supplementary Legends. [file 41598_2024_52956_MOESM11_ESM.docx]

**SUPPLEMENTARY MATERIAL CAPTIONS**

**Supplementary Figure 1.** Clinical characteristics in the original cohort. (A), (B), (C), (D) The violin plot of the clinical characteristics of patients. Component comparison was conducted using Welch's one-way ANOVA and Games-Howell test. ND, blue; IGT, orange; DEP, green.

**Supplementary Figure 2.** Correlation of immunocyte infiltration in the pancreatic islet microenvironment of DEP patients (*p < 0.05; **p ≤ 0.01)

**Supplementary Figure 3.** The clinical characteristics and the sum of normalized score in the restricted cohort. (A), (B), (C), (D) The violin plot of the clinical characteristics between different diabetes status. ND, blue; IGT-A, darkcyan; IGT-B, darkorange; DEP, green. (E), (F), (G), (H) Correlation be-tween the clinical parameters and the sum of normalized score in the restricted cohort. Pearson’s correlation coefficient was used to check the association. The grey line is the fitted curve.

**Supplementary Figure 4.** The normalized score for different immunocytes across the pairwise group in the GSE164416 dataset. Component comparison using Student’s t-test and the Games-Howell test (NS, p > 0.05; *p < 0.05; **p ≤ 0.01; ***p ≤ 0.001).

**Supplementary Figure 5.** The normalized score for different immune cells across the pairwise group. (A), (B), (C) The split violin plot of the normalized score for different immune cells across the pairwise group. Component comparison using Student’s t-test and the Games-Howell test (NS, p > 0.05; *p < 0.05; **p ≤ 0.01; ***p ≤ 0.001).

**Supplementary Figure 6.** The receiver operating characteristic (ROC) curves for the clinical characteristics and B2M of DP patients. (A) The ROC curves in the GSE164416 dataset. (B) The ROC curves in the GSE76895 dataset.

**Supplementary Figure 7.** The expression of biomarkers in the independent cohort. (A) The relative abundance of cells based on marker genes in the GSE76895 cohort. (B) The violin plot of the expression of B2M from the animal models. (C) The violin plot of the expression of B2M from the patient tis-sue. (D) The immunohistochemical results of B2M between the normal tissue and the adenocarcinoma tissue.

**Supplementary Table 1.** Baseline clinical characteristic of patients in the GSE164416 dataset.

**Supplementary Table 2.** Baseline clinical characteristic of patients in the GSE76895 dataset.

**Supplementary Table 3.** Difference analysis results across pairwise group comparisons.
