# Supplementary material for: Mechanism exploration and biomarker identification of glycemic deterioration in patients with diseases of the exocrine pancreas
Source: Sci Rep. 2024 Feb 22;14:4374. doi: 10.1038/s41598-024-52956-x (PMC10883946; doi:10.1038/s41598-024-52956-x)
Supplement: Supplementary file 12 — Supplementary Information 1. [file 41598_2024_52956_MOESM12_ESM.pptx]

## Slide 1
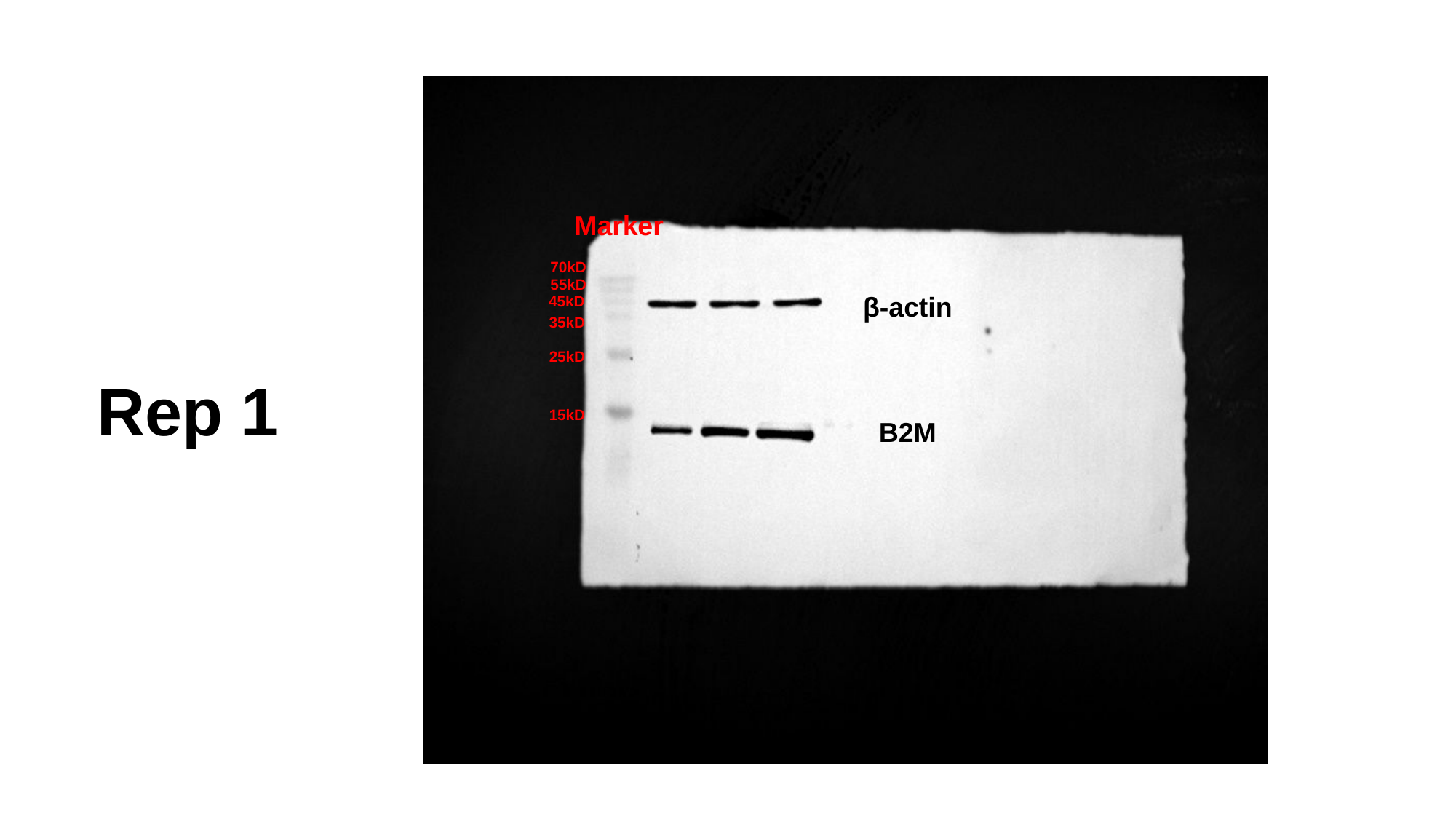

Marker
70kD
55kD
β-actin
45kD
35kD
25kD
Rep 1
15kD
B2M

## Slide 2
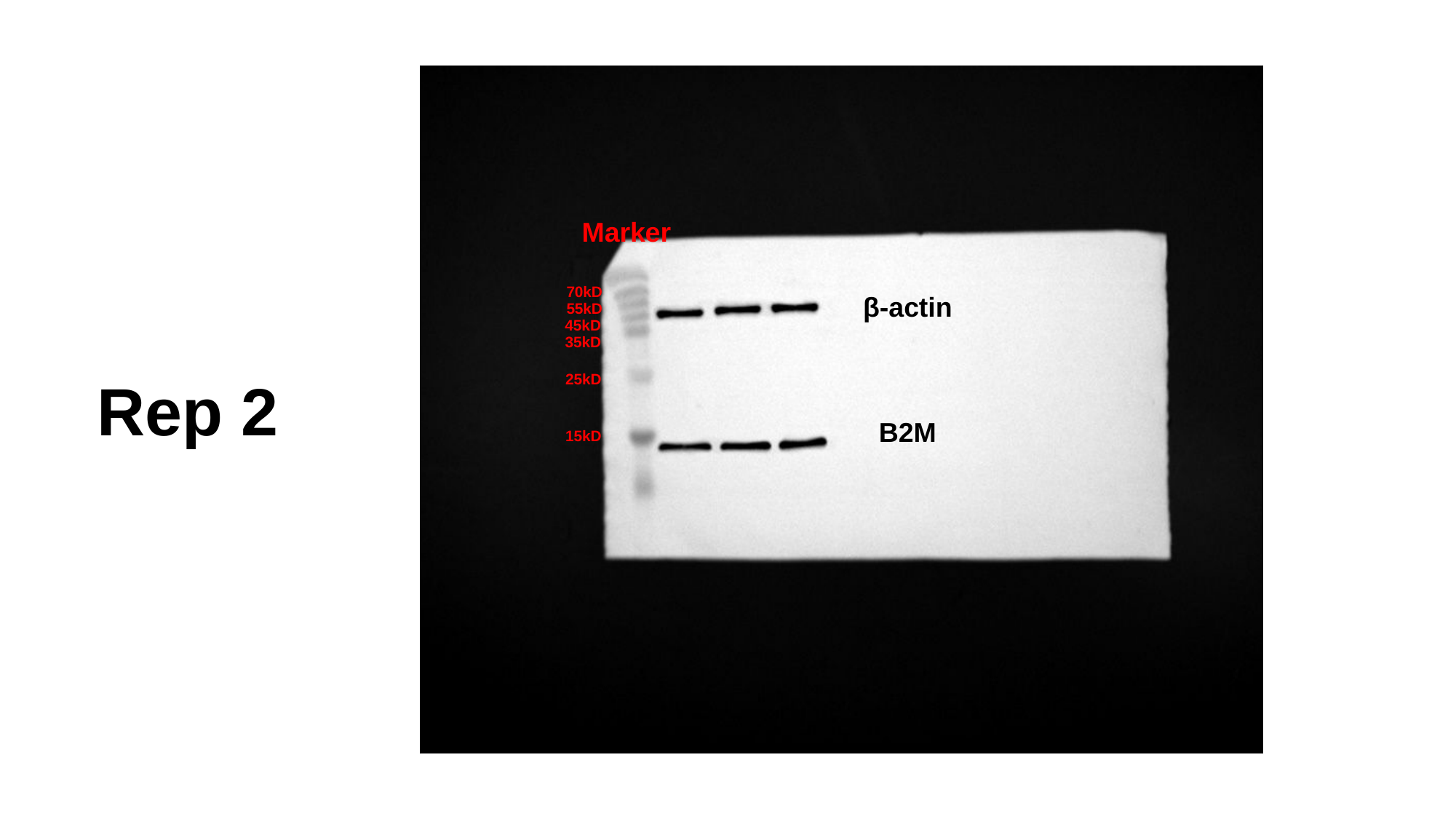

Marker
70kD
β-actin
55kD
45kD
35kD
Rep 2
25kD
B2M
15kD

## Slide 3
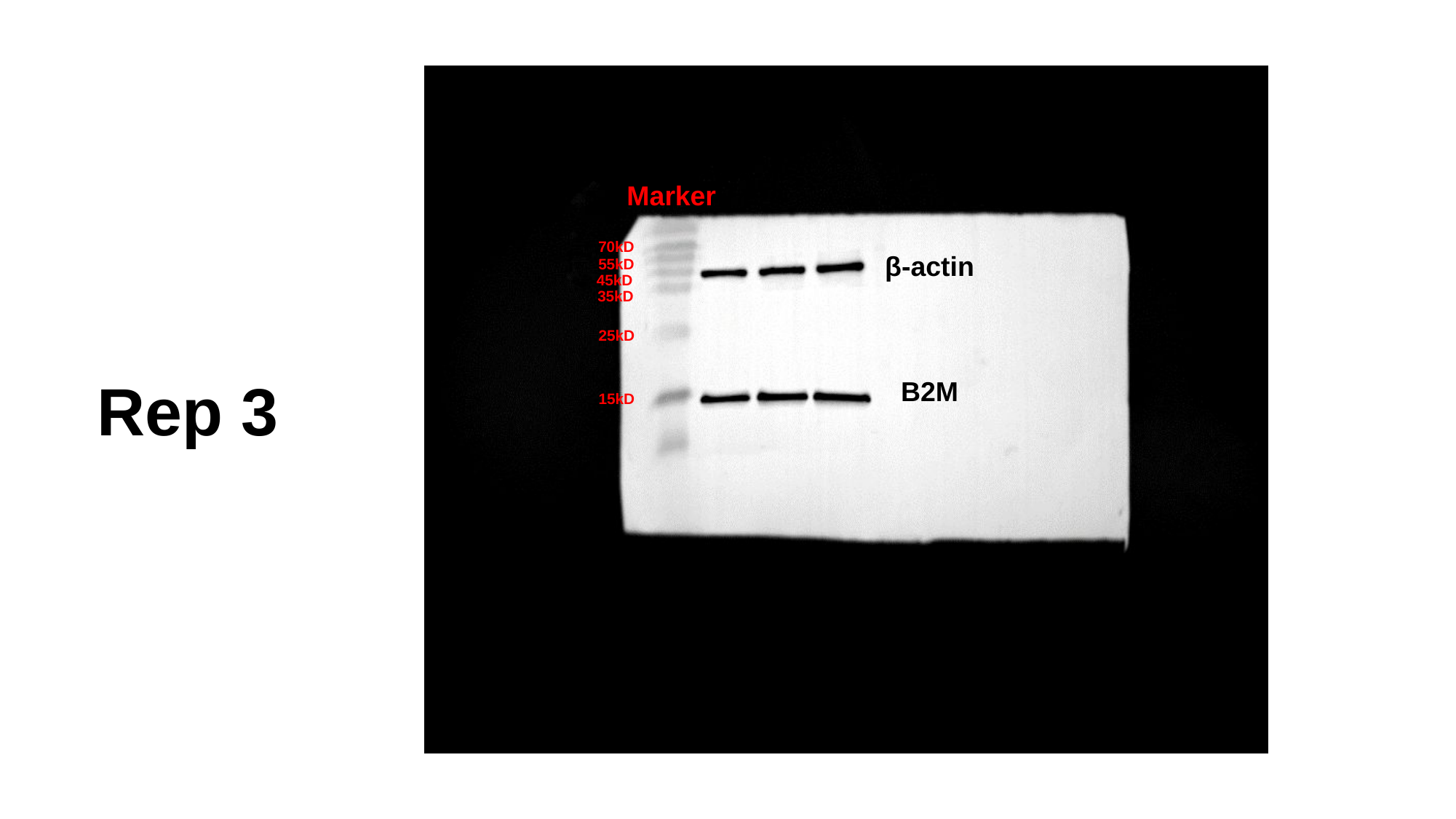

Marker
70kD
β-actin
55kD
45kD
35kD
25kD
Rep 3
B2M
15kD
